# Long-term patient-derived ovarian cancer organoids closely recapitulate tumor of origin and clinical response

**DOI:** 10.1186/s13046-025-03537-x

**Published:** 2025-10-07

**Authors:** Lucie Thorel, Enora Dolivet, Pierre-Marie Morice, Romane Florent, Jordane Divoux, Marion Perréard, Lucie Lecouflet, Guillaume Desmartin, Chloé Marde Alagama, Florence Giffard, Alexandra Leconte, Justine Lequesne, Bénédicte Clarisse, Mélanie Briand, Alimatou Traoré, Céline Villenet, Jean-Pascal Meneboo, Guillaume Babin, Léopold Gaichies, Sandrine Martin-Françoise, Jean-François Le Brun, Roman Rouzier, Emilie Brotin, Christophe Denoyelle, Nicolas Vigneron, Raphaël Leman, Dominique Vaur, Laurent Castera, Cécile Blanc-Fournier, Nicolas Elie, Benoit Plancoulaine, Florence Joly, Matthieu Meryet-Figuière, Martin Figeac, Louis-Bastien Weiswald, Laurent Poulain

**Affiliations:** 1 Université de Caen Normandie, INSERM U1086 ANTICIPE (Interdisciplinary Research Unit for Cancers Prevention and Treatment), BioTICLA Laboratory (Precision Medicine for Ovarian Cancers), Caen, France; 2 UNICANCER, Comprehensive Cancer Center François Baclesse, Caen, France; 3 UNICANCER, Comprehensive Cancer Center François Baclesse, Department of Surgery, Caen, France; 4 Université de Caen Normandie, PLATON Services Unit, ORGAPRED Core Facility, Caen, France; 5 Université de Caen Normandie, PLATON Services Unit, VIRTUAL’HIS Core Facility, Caen, France; 6 UNICANCER, Comprehensive Cancer Center François Baclesse, Clinical Research Department, Caen, France; 7 Université de Caen Normandie, PLATON Services Unit, Biological Resource Center ‘OvaRessources’, Caen, France; 8 University of Lille, CNRS, Inserm, CHU Lille, Institut Pasteur de Lille, US 41 – UAR 2014 – PLBS, Lille, France; 9 Université de Caen Normandie, PLATON Services Unit, ImpedanCELL Core Facility, Caen, France; 10 UNICANCER, Comprehensive Cancer Center François Baclesse, Calvados General Tumor Registry, Caen, France; 11 UNICANCER, Comprehensive Cancer Center François Baclesse, Laboratory of Biology and Cancer Genetics, Caen, France; 12 Normandie Université, INSERM U1245, Cancer Brain and Genomics, FHU G4 Génomique, Rouen, France; 13 UNICANCER, Comprehensive Cancer Center François Baclesse, Department of Bio-Pathology, Caen, France; 14 Vilnius University, Institute of Biomedical Sciences, Faculty of Medicine, Forensic Medicine and Pharmacology, Department of Pathology, Vilnius, Lithuania

**Keywords:** Ovarian cancer, Patient-derived tumor organoids, Homologous recombination, Functional assay

## Abstract

**Background:**

Ovarian cancers are the second cause of death from gynecological cancers worldwide, due to a late diagnosis combined with the development of resistance to chemotherapy. However, half of these cancers present alterations in Homologous Recombination (HR), making them sensitive to inhibitors of the PARP protein (PARPi), involved in DNA repair. Nevertheless, identifying patients who respond to chemotherapy and selecting those eligible for PARPi remains a challenge for clinicians. In this context, the use of Patient-Derived Tumor Organoids (PDTO) for predictive functional testing represents an interesting prospect for clinical decision making.

**Methods:**

Here we established a panel of 37 long-term PDTO models of various histological subtypes from 31 ovarian cancer patients. Histological and molecular profiles of PDTO were compared to tumor sample of origin using immunohistochemical analyses and global approaches (copy number variation and transcriptomic profiling). PDTO models were exposed to standard drugs for ovarian cancer patients, including PARPi, and response was assessed using viability assay. To further define the HR status of PDTO, we performed a functional assay evaluating the ability of PDTO to initiate HR (RECAP test) using automated histo-imaging quantitative analysis of RAD51 foci, as well as an NGS analysis based on the sequencing of an HR-related genes panel to obtain a Genome Instability Score (GIS).

**Results:**

We demonstrated that PDTO mimicked histological and expression of tumor markers of paired tumors. Moreover, non-negative matrix factorization approach revealed that PDTO recapitulated the transcriptomic profile of the cancer component from their sample of origin. Screening of chemotherapeutic drugs showed that PDTO exhibit heterogeneous responses, and that response of PDTO from high-grade serous ovarian carcinoma to carboplatin recapitulated patient response to first-line treatment. Additionally, the detection of HRD phenotype of PDTO using functional assay was associated with the results of the HRD test Genomic Instability Scar (GIScar).

**Conclusion:**

Although larger-scale investigations are needed to confirm the predictive potential of PDTO, these results provide further evidence of the potential interest of ovarian PDTO for functional precision medicine.

**Supplementary Information:**

The online version contains supplementary material available at 10.1186/s13046-025-03537-x.

## Background

Ovarian Cancer (OC) is the 8th most common cancer in women worldwide, with approximately 325,000 new cases and 207,000 deaths in 2022 [1]. Epithelial OC represent 95% of OC and are a heterogeneous disease. Based on histopathology and molecular genetic alterations, they are divided into 5 major subtypes with distinct biological and molecular properties: high-grade serous, low-grade serous, clear cell, endometrioid, and mucinous ovarian carcinomas [2]. Due to the usually asymptomatic nature of early stages, most of OC cases are diagnosed at late stages (e.g. stage III/IV as determined by the International Federation of Obstetrics and Gynecology (FIGO)) when cancer has spread widely to the peritoneal cavity [3]. The standard of care consists of primary or interval cytoreductive surgery followed by combination platinum-taxane chemotherapy with or without bevacizumab [4]. However, around 70% of patients with OC will recur after first-line therapy, leading to a 5-year relative survival rate of only 29% for late stages [5]. Thus, it is well known that a “one-size-fits-all” treatment approach should no longer be applied. The introduction of targeted maintenance therapy with poly ADP-ribose polymerase inhibitors (PARPi) to the first-line setting has led to remarkable improvement in outcome in selected patients, especially those with tumors harboring *BRCA1/2 (breast cancer genes 1 and 2)* mutations and Homologous Recombination Deficiency (HRD) [6]. At time of recurrence, according to the guidelines, the choice of treatment regimen will be based on platinum sensitivity, prior lines of treatment, chemotherapy-related adverse events, *BRCA* mutation status and physician and patients’ preferences [4]. In the case of early recurrence, the options of treatment are limited, with a response rate of less than 15% [2], highlighting the lack of predictive biomarkers for guided anticancer agent selection. This also applies to PARPi, since the sensitivity to PARPi is not restricted to tumors harboring *BRCA1/BRCA2* mutations [7]. Therefore, a range of genetic tests are being developed to improve profiling of HR status, including the academic HRD test Genomic Instability Scar (GIScar) [8].

However, genomics precision medicine has several limitations, such as the lack of selectivity of some molecular signatures [7], the limits of the interpretation (complex mutational signatures or variants of unknown significance) [9] and the fact that genomic instability signatures inform on a history of instability. Moreover, it does not warrant that such instability is still present.

This underscores the interest in developing functional precision medicine, an approach based on direct drug exposure of patient-derived tumor models to predict patient response [10]. Improved feasibility of generating preclinical models mimicking the patient tumor, such as patient-derived tumor organoids (PDTO), has made these models accessible for personalized treatment. PDTO are 3D multicellular structures generated from patient tumor cells embedded in basement membrane matrix and cultured in a medium supplemented with a cocktail of cell signaling pathways activators and inhibitors to reproduce in vivo niche conditions and allow long term growth. PDTO faithfully recapitulate the histological and molecular characteristics of the tumor from which they are derived. They can be rapidly expanded and established from a small sample size such as needle biopsy with higher success rate compared to other models [11]. Most of all, a growing body of evidence indicates that PDTOs are able to predict the clinical response, although most of clinical studies were based on small sample size (< 10 patients) [12, 13]. PDTO cultures have already been successfully established from OC patients with success rate of establishment ranging from 18 to 90% but with varied definitions of an established model [14–22]. However, in most of the studies, the success rate of establishment as well as long-term stable passages are usually not explicitly mentioned in the literature [23]. Interestingly, several of them demonstrated high level of correlation between response of PDTO and clinical response despite the small sizes of samples, ranging from 1 to 7 patients [18, 20–22].

In this study, we established and performed a comprehensive characterization of a panel of 37 long-term PDTO derived from 31 patients with various OC subtypes and determined whether response of PDTO to first-line chemotherapy correlates with patient response to assess clinical relevance of these models.

## Methods

### Patient-derived tumor organoid establishment

***Ethical considerations***. Fresh tumoral tissue and ascitic fluids from ovarian cancers were collected from patients treated at the Comprehensive Cancer Center François Baclesse (Unicancer Center, Normandy). Informed consent forms were signed by all patients and were obtained either by the Biological Resources Center ‘OvaRessources’, which has received NF 96 900 accreditation (N° 2016/72860.1) or in the context of the ‘OVAREX’ clinical trial (N°ID-RCB: 2018-A02152-53, NCT03831230) [24], in accordance with ethical committee and European law. Clinical, treatment and histopathological details were extracted from patient charts. A medical pathologist analyzed all samples.

***Processing of samples***. Fresh samples were processed as previously described [25]. Briefly, tumor tissue was cut into 4 mm^3^ pieces. One piece was fixed in 3% paraformaldehyde for paraffin embedding and subsequent histopathological/immunochemistry analyses, two pieces were snap frozen in FlashFreeze (Milestone) and stored at -150 °C for DNA/RNA extractions, and another piece was processed to establish PDTO. Tumor sample dissociation was performed using the Tumor Dissociation human kit and a gentleMACS Dissociator according to the manufacturer’s instructions (Miltenyi Biotec). Sterile tumor ascitic samples were centrifuged (430 g for 5 min). Pellets containing cells were resuspended in 20 mL of RPMI 1640 medium (Fisher Scientific) supplemented with 10 UI/mL penicillin, 10 µg/mL streptomycin (Fisher Scientific) and 1% Bovine Serum Albumin (BSA) (Sigma). Suspensions were strained successively in 300 μm and 50 μm filters (Endecotts). Remaining cells or aggregates were digested in 2 mL of TrypLE Express (Fisher Scientific) at 37 °C up to 15 min. Dissociated cells from tumors and ascites were collected in organoid basal medium [OBM: Advanced DMEM (Fisher Scientific), 10 UI/mL penicillin, 10 µg/mL streptomycin, 1% GlutaMAX-1 (Fisher Scientific)] and pelleted (430 g for 5 min). 10 000–20 000 cells were resuspended in organoid culture medium (OBM supplemented with B27 (Fischer Scientific, 200 µL/mL), N-Acetyl-L-cysteine (Sigma, 1.25 mM), EGF (Miltenyi, 50 ng/mL), FGF-10 (Peprotech, 20 ng/mL), FgF-basic (Miltenyi, 1 ng/mL), A-83-01 (Peprotech, 500 nM), Y27632 (Selleckchem, 10 µM), SB202190 (Peprotech, 1 µM), Nicotinamide (Sigma, 10 mM), PGE2 (Sigma, 1 µM), Primocin (InvivoGen, 100 µg/mL), cultrex HA-R-Spondin-1-Fc 293T (AMS Bio, 50% V/V) and Cultrex L-WRN (AMS Bio, 10% V/V), mixed with 70% Cultrex Reduced Growth Factor Basement Membrane Extract, Type 2 (BME2) and seeded in a pre-warmed 24-well plate (Eppendorf). After polymerization (37 °C, 5% CO2, 15 min), each drop was immersed with 500 µL of organoid culture medium. Medium was exchanged twice a week using automated liquid handling workstation (Microlab STAR line, Hamilton). Once harvested with OBM supplemented with 1% BSA (OBM-BSA), PDTO were dissociated using TrypLE Express (37 °C up to 10 min). Isolated cells were seeded or biobanked (Coolcell, -80 °C) in 500 µL of Recovery cell culture freezing medium (Fisher Scientific) for future use.

***PDTO culture***. When PDTO reached around 150 μm in diameter, they were collected with cold OBM-BSA, centrifuged at 200 g for 2 min and incubated with TrypLE Express for up to 15 min at 37 °C. After dissociation, cells were centrifuged at 430 g for 5 min, suspended in organoid culture medium, counted and plated at 10 000 cells per 50 µl drop of 70% BME2 in pre-warmed 24-well plates. Plates were transferred to a humidified 37 °C/5% CO2 incubator. Cryovials were prepared at regular intervals by dissociating and resuspending PDTO in Recovery Cell Culture Freezing Medium (Gibco), then placed in a cell freezing container (Coolcell) at -80 °C and biobanked at -150 °C on the next day. PDTO lines were authenticated by comparison of their short tandem repeat (STR) profiles with that of tumor sample of origin (Microsynth).

### Characterization

***Histology and immunohistochemistry***. Tissue and PDTO were fixed in 3% paraformaldehyde overnight. After the embedding of PDTO in 2% agarose, samples were dehydrated, paraffin embedded, and sectioned before standard haematoxylin and eosin staining (H&E). Automated immunohistochemistry using a Ventana Discovery Ultra (Roche) was performed on 4 µm-thick paraffin sections. Slides were deparaffinized with EZPrep buffer and epitopes were unmasked by 56 min of high-temperature treatment in CC1 EDTA buffer. Sections were incubated for 40 min at 37°C with an anti PAX8 (ab191870, Abcam, 1/500), p53 (ab16665, Abcam, 1/100), WT1 (ab89901, Abcam, 1/300), Napsin A (ab133249, Abcam, 1/1000), HNF1β (ab213149, Abcam, 1/2000) or Ki67 antibody (NCL-Ki67p, Novocastra, 1/500). Secondary antibody (Omnimap Rabbit HRP; Ventana Medical System Inc., Tucson, AZ, USA) was incubated for 16 min at room temperature. Immunodetection performed without the primary antibody was used as control. After washes, the staining was performed with DAB (3, 3’-diaminobenzidine) and sections were counterstained with hematoxylin using Ventana reagents according to the manufacturer’s protocol. Stained slides were then digitized with a 20X magnification using the Vectra Polaris slide scanner (Akoya Biosciences).

### Genomic and transcriptomic analyses

***DNA and RNA extraction***. DNA and RNA extractions were performed using the NucleoSpin RNA and tissue kits according to the manufacturer protocol (Macherey–Nagel). After extraction DNA and RNA samples were stored at -80 °C.

***Low pass WGS***. Low Pass WGS was performed on DNA samples. Library was prepared using Illumina DNA PCR-Free Prep (Illumina) starting from 500 ng of DNA input (except for 6 samples: 93, 356, 384, 401, 430 and 483ng). Libraries were pooled all together to be sequenced on one flowcell. 150 bp paired-end sequencing of the samples was performed on the NovaSeq 6000 (Illumina). Raw reads were mapped to the human reference genome (GRCh38) using the Burrows-Wheeler Aligner (BWA) with MEM algorithm (version 0.7.17). Read duplicates were removed by Picard MarkDuplicates (version 2.27.5). Read count was performed by HMMcopy (version 0.1.1) with a bin (non-overlapping window) of 50 kb. Copy number alteration (CNA) identification and tumor fraction estimation were performed using ichorCNA (version 0.3.2). For data visualization, R/Bioconductor packages karyoploteR (version 1.16.0) and copynumber (version 1.30.0) were used.

***Transcriptome***. Starting from 4 µl of total RNA we add 1 µl of ERCC spike-in control. Library generation is then initiated by oligo dT priming, from total RNA (200 ng RNA input in 4 µl) following Lexogen QUANTSEQ FWD + UMI protocol. After first strand synthesis the RNA is degraded and second strand synthesis is initiated by random priming and a DNA polymerase. At this step Unique Molecular Identifiers (UMIs) are introduced allowing the elimination of PCR duplicates during the analysis. After obtaining the double stranded cDNA library, the library is purified with magnetic beads and amplified. During the library amplification, the barcodes and sequences required for cluster generation (index i7 in 3’ and index i5 in 5’) are introduced thanks to Illumina-compatible linker sequences with 14 cycles of amplification. The final library is purified and checked on a High sensitivity DNA chip to be controlled on the Agilent bioanalyzer 2100. Each library is pooled equimolarly and the final pool is also controlled on Agilent bioanalyzer 2100 and sequenced on NovaSeq 6000 (Illumina) (100 bp single-end).

***Data analysis for transcriptomic data***. To eliminate poor quality regions and poly(A) of the reads, we used the fastp program. We used quality score threshold of 20 and removed the reads shorter than 25 pb. The read alignments were performed using the STAR program with the human genome reference (GRCh38) and the reference gene annotations (Ensembl). The UMI (Unique Molecular Index) allowed to reduce errors and quantitative PCR bias using fastp and UMI-tools. Based on read alignments, we counted the number of molecules by gene using FeatureCount. Others programs were performed for the quality control of reads and for the workflow as qualimap, fastp, FastQC and MultiQC. Differential Gene Expression of RNA-seq was performed with R/Bioconductor package DESeq2.

***NMF on transcriptomic data***. Non-negative Matrix Factorization (NMF) was applied using the butchR package. We started from a matrix of normalized expressed genes (13 401 expressed genes) and applied variance stabilizing transformation (VST). We started to apply NMF on a subset of the VST matrix subsetting to PDTO samples only. For each analysis different rank (k) were tested (from 2 to 18). Then we projected the NMF factors on all samples (tumors and PDTO) using the LCD function from the R YAPSA package using as input the count matrix (VST) and the W matrix from the NMF applied on PDTO samples. Given the NMF on all samples, we plotted UMAP projection using ggplot and computed distance between paired samples (PDTO/tumor) based on the UMAP. To choose the best rank, we choose the one that minimize the sum of distances between paired samples (PDTO/tumor). We then fixed the rank to k = 12. Each NMF-derived signature corresponds to a latent transcriptional program characterized by a specific gene weight distribution. The top 5% most contributing genes for each signature are provided in Supplementary Data File 1.

### Evaluation of response to treatments

***Drugs***. After reconstitution in saline solution, doxorubicin (Teva) and gemcitabine (Sandoz) were stored at 4 °C, paclitaxel and carboplatin (Accord) were stored at room temperature. Olaparib and niraparib (Medchemexpress) were diluted in DMSO and stored as stock solution at -80 °C.

***PDTO treatment***. When PDTO reached the size of 75–150 μm in diameter, PDTO were collected with cold OBM-BSA and centrifugated at 200 g for 2 min. PDTO were resuspended in organoid treatment medium (organoid culture medium lacking primocin, Y-27632 and N-acetylcysteine) and counted. For treatment in 96-well plates, PDTO were resuspended in 2% BME2/organoid treatment medium and 200 PDTO per well were seeded in 100 µL volume in a previously coated (1:1 organoid treatment medium/BME2) white clear bottom 96-well plates (Greiner). For treatment in 384-well plates, PDTO were resuspended in 10% BME2/organoid treatment medium and 50 PDTO per well were seeded in 20 µL in black ULA clear bottom 384-well plates. Thirty minutes later, PDTO were exposed to treatments and PDTO morphology was monitored using CellDiscoverer 7 (Zeiss). One week later, ATP levels were measured by CellTiter-Glo 3D assay (Promega) and luminescence was quantified using GloMax (Promega). Cell viability values were normalized to control and treatment sensitivity was expressed as the average of at least two independent biological replicates. Positive control (Staurosporine 10 µM) was used to calculate a z-score as followed:

$$\displaylines{ z - score = 1 - \cr \frac{{3*{standard}\,{deviation}\left( {negative}\,{control} \right) + 3*{standard}\,{deviation}\left( {positive}\,{control} \right)}}{{average\left( {negative}\,{control} \right) - average\left( {positive}\,{control} \right)}} \cr} $$

An experiment with a z-score under 0.4 was considered as non-exploitable. Viability curves were designed using GraphPad Prism software (version 9.2.0). The area under the curve (AUC) was computed for each PDTO model. Normalized AUC z-scores were calculated by subtracting the mean and divided the results by the standard-deviation of the AUC compared to each AUC value.

**Genomic instability scoring of PDTO**. In order to assess PDTO homologous recombination (HR) status, PDTO were sequenced with a 127-genes panel including 15 HR genes (*BRCA1*, *BRCA2*, *ATM*, *BARD1*, *BRIP1*, *CDK12*, *CHEK1*, *CHEK2*, *FANCL*, *PALB2*, *PPP2R2A*, *RAD51B*, *RAD51C*, *RAD51D*, *RAD54L*). Following sequencing and alignment using BWA (0.7.17-r1188), variant calling was performed using a combination of algorithms: HaplotypeCaller (4.1.6.0), VarDict (v1.8.2), and Mutect (v2.2). Only variants with a variant allele frequency (VAF) ≥ 5% were considered for downstream analysis. Mutations were reported if they were either classified as known oncogenic variant according to the ONCOKb and ClinVar databases, or if they were predicted to be inactivating (e.g., frameshift mutations, nonsense mutations, or alterations affecting consensus splice sites). The sequencing data were then be used to determine a Genomic Instability Score (GIS) as described by Leman et al. [8]. Briefly, this genomic instability score was based on a first analysis by a CNVkit pipeline v0.9.7 [26]. From the CNVkit data outcome, three scores of instability were calculated: the number of large genomic events, the structural instability score, and the allelic imbalance. Then, these scores were used in the logistic model to compute a mathematical esperance that the tumor is HR-proficient (HRP) or HR-deficient (HRD), with a value tending to 0 for HRP tumors and a value tending to 1 for HRD tumors. The decision-making threshold between HRP and HRD tumors was 0.48. However, this threshold was defined on FFPE tumor tissue and at the time of this work we could not propose an optimal threshold for PTDO. We defined so a third category HRDmid corresponding to PTDO with a score at more and less 0.25 than the threshold of FFPE tumor tissues (0.48), i.e. a range score of [0.23–0.73].

### RECAP test

***PDTO irradiation***. To evaluate the homologous recombination deficiency status, we used the RECAP test described by Naipal and al. [27]. PDTO were grown in BME-2 domes in 6-well plates. When PDTO reached the size of 75–150 μm in diameter the medium was collected and conserved at 37 °C. Two ml of OBM were added to each well (including control plate) before PDTO were irradiated at 5 Gy (X-Ray source, 130 kV, 5 mA) in a CellRad X-irradiation system. After irradiation, the medium was discarded and replaced by the previously organoid culture medium conserved at 37 °C. Following incubation of the plates for 2 h, 6–24 h, PDTO were collected, washed with OBM-BSA and fixed in 3% PFA for at least 3 h at + 4 °C. PFA was then discarded and the pellet was embedded in agarose, dehydrated and paraffin-embedded (Autostainer XL, Leica).

***PDTO staining***. Immunofluorescence staining of RAD51 (ab133534, Abcam) and Cyclin A2 (ab211736, Abcam) were done on 4 μm sections of PDTO (microtome Leica) using Ventana Discovery Ultra (Roche). Microscope slides with PDTO sections were deparaffinized with EZPrep buffer and the antigenic sites were unmasked with EDTA. The endogen peroxidase inhibitor (Roche) was used to limit to background noise. 100 µl of primary antibody previously diluted (Primary Antibody Diluant, Clinisciences) were added to each slide and incubated at 37 °C for 40 min (1/100 000 for RAD51 and 1/1 000 for Cyclin A2). Then, after washing, the second antibody (Omnimap Rabbit) was incubated at 37 °C for 16 min. Finally, the DISCOVERY Rhodamin (Roche) and the DISCOVERY FITC (Roche) were added for RAD51 and Cyclin A2, respectively. The slides were subsequently stained for DAPI (Roche). At the end of the procedure, the slides were washed with detergent to eliminate the LCS oil layer and were hydrophilic-mounted with Fluoromount-G (CliniSciences). The slides were protected from light until digital acquisition.

***Digital acquisition***. Specimens of PDTO sections were digitized with an Olympus VS120 scanner equipped with a 40x objective (N.A 0.95), a LED illumination (Lumencor Spectra X 7 LED), a single multiband filter DAPI (emission: 455 nm; exposure time: 5ms), FITC (518 nm; 20ms) and CY3 (565 nm; 10ms) filters from Olympus and a CMOS camera from Hamamatsu (Orca Flash 4.0). The whole slide images (WSI) were recorded with Extended Focal Imaging (EFI) technology applied over a height of 4 μm (Z-range) and an acquisition of 5 slices per field (0.84 μm Z-spacing).

***Quantification processing***. On each slide, the PDTO were isolated automatically by detection with Qupath program and the extension Cellpose [28, 29]. For each individual PDTO, nuclei detection was assessed on the DAPI channel and the surface occupied by nuclei was processed using extension Stardist of Qupath program [30]. For the positive nuclei detection of Cyclin A2, two Gaussian filters of different sizes were applied on the FITC channel. Only the positive pixels obtained after the difference between the two filtered images were kept to obtain a binary image with the positive Cyclin A2 areas. Then a geodesic reconstruction was applied between the images of nuclei and Cyclin A2 to identify only positive nuclei with a Cyclin A2 active. Finally, for the detection of RAD51 foci, the CY3 channel was used. RAD51 foci were detected thanks to a Laplacian filter after a Gaussian operation, in the scikit-image python library [31]. Only bright foci on dark backgrounds are detected. For each nucleus of each organoid, only the RAD51 foci inside the CyA2-positive nuclei were kept and computed.

***Gaussian Mixture Model approach***. A Gaussian Mixture Model (GMM) [32] was used to determine the minimum threshold of RAD51 foci in proliferative cells (Cyclin A2-positive nuclei), making it possible to differentiate the basal level of HR DNA repair from irradiation-induced HR DNA repair. Ultimately one Gaussian function was found but it allowed the smoothing of the histograms. Three different indexes were described using the gaussian approach: the damage index (DI) equal to the mean of the control gaussian, the radio-induced damage index (RDI) the difference between the mean of the 2 h gaussian and the mean of the control gaussian, and the reparation index (RI) the difference between the mean of the 24 h gaussian and the mean of the 2 h gaussian. The indexes were computed for each PDTO model and Principal Component Analysis (PCA) was applied to keep only principal components (PC) with maximum variance [33]. A cutoff value of -0.5 was used on the PC1 axis to categorize the homologous recombination status, with the negative value characterizing HRD status and the positive values HRP ones.

### Statistics

Quantitative variables were compared with unpaired two-tailed Mann-Whitney test using GraphPad Prism. Correlations between gene expression levels of PDTO and tumor were calculated using Pearson’s R. Survival were calculated using the Kaplan-Meier method, and survival distributions were compared using the Log-Rank test. *P* values < 0.05 were considered significant. Definitions of *n* and details of statistical analyses are provided in relevant figure legends.

## Result

### Establishment and characterization of long-term ovarian PDTO

**Fig. 1:**
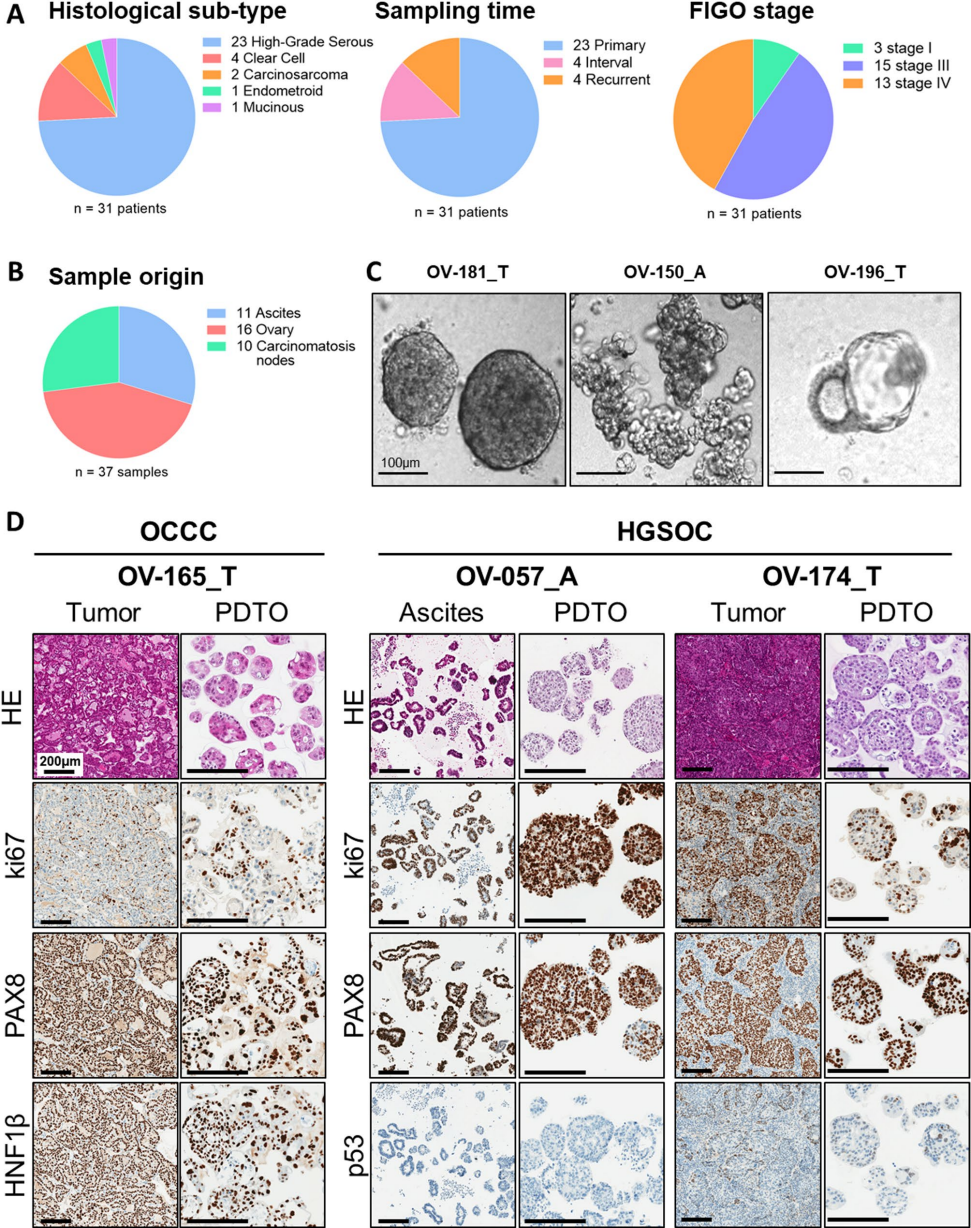
Establishment and characterization of a PDTO panel from ovarian tumors. (**A**) Pie-chart showing the histological subtype, sampling time, sample origin and FIGO stage of the 31 patients from which PDTO lines were established. (**B**) Pie-chart showing the sample origin of the 37 ovarian PDTO lines. (**C**) Representative pictures of PDTO of various morphologies. A = Ascites-derived PDTO and T = Tumor–derived PDTO. Scale bar = 100 μm. (**D**) Hematoxylin and Eosin (HE) staining and immunohistochemistry analysis of ovarian cancer protein markers ki67, PAX8, p53 and HNF1β in tumor tissues and paired PDTO. Scale bar = 200 μm

Long-term PDTO were considered established as PDTO line after cell expansion over 8 successive passages and the ability to grow in culture after thawing (13.8% success rate, *n* = 224, Table S1). No failure in thawing and regrowing PDTO lines that had successfully expanded over 8 successive passages was observed. We generated 37 ovarian cancer long-term PDTO from 31 patients of various histotypes, with a majority of high-grade serous ovarian cancer (HGSOC, 23/31) but also rarer subtypes, including ovarian clear cell carcinomas (OCCC, 4/31), carcinosarcoma (CS, 2/31), endometrioid (EM, 1/31) and mucinous (MC, 1/31) (Fig. 1A).

Out of 37, 8 PDTO were established from pretreated patients. All of these 8 PDTO models were generated from 8 independent patients with HGSOC or CS including 4 from an interval surgery (after 3 chemotherapy cycles) and 4 from ascites received after 2 or more treatment lines (Fig. 1A). Moreover, since OC is often diagnosed at a late stage, most of the PDTO lines were derived from stage FIGO III and IV (28/31). Stage FIGO I (3/31) are exclusively derived from mucinous and clear cell carcinomas (Fig. 1A). Samples were mainly obtained from solid tumor (26/37 established models, including 16 ovary and 10 carcinomatosis nodes) but PDTO establishment from ascitic samples was also successful (11/37 established models) (Fig. 1B). More patient clinical data are presented in Table S2.

Ovarian PDTO models displayed heterogeneous phenotypes, such as dense morphology for the majority of cases (Fig. 1C, left panel), grape-like (Fig. 1C, middle panel), or cystic morphologies (Fig. 1C, right panel). In order to compare ovarian PDTO with their tumor of origin, Hematoxylin and Eosin (HE) staining was performed, as well as immunostaining of OC diagnosis proteins including PAX8, p53 and a marker of proliferation Ki67. For OCCC, additional staining of HNF1β and Napsin A was achieved in order to better characterize this specific subtype (Fig. 1D, Fig. S1) [34]. A strong concordance between the original tumors and PDTO was observed. However, WT1 expression was reduced in some HGSOC-derived PDTO compared to the corresponding tumors (Fig. S1). Overall, PDTO retained the morphological features of their original tumor, as well as the expression profile of ovarian cancer diagnosis proteins.

### Ovarian PDTO recapitulate molecular landscape of tumors

**Fig. 2:**
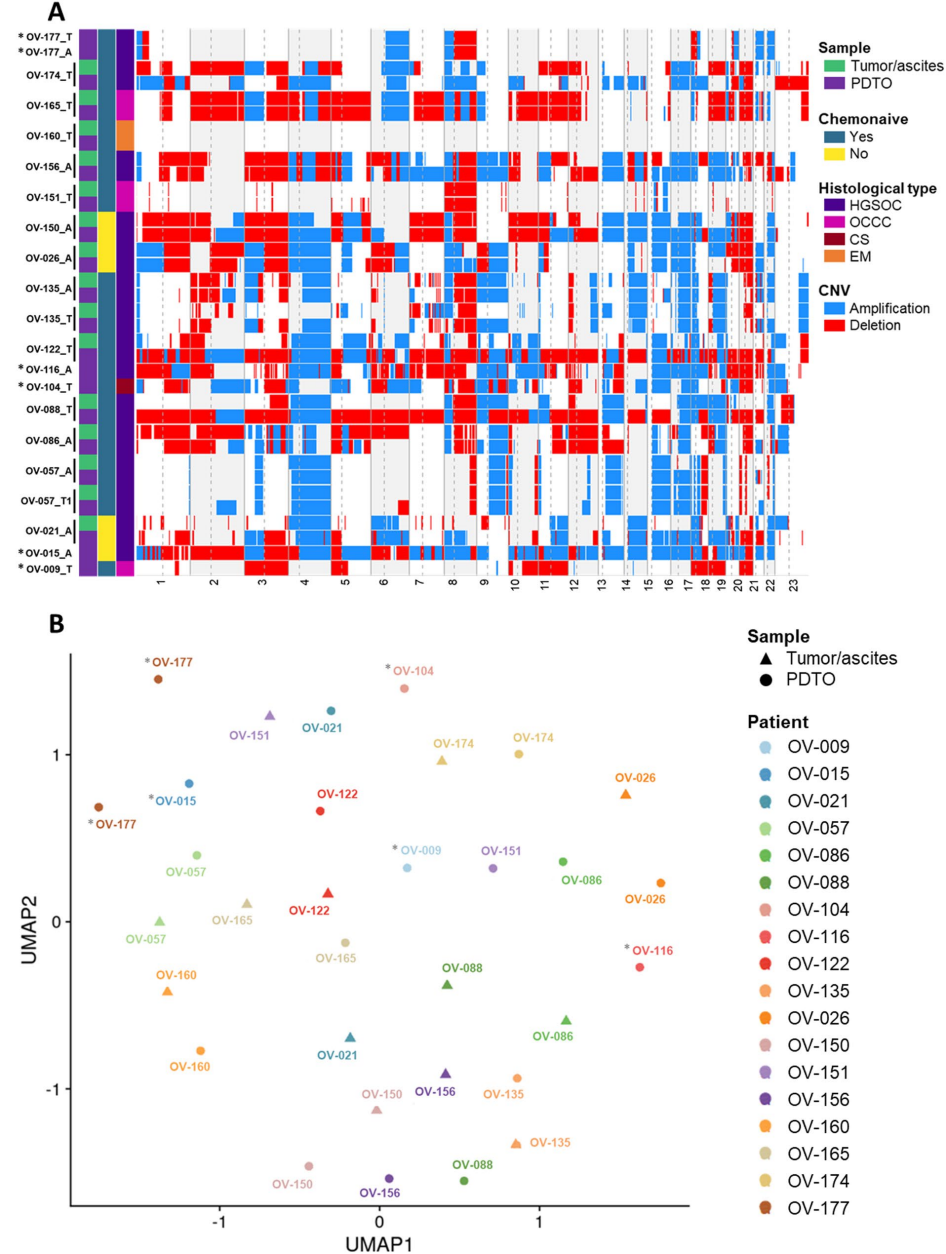
Ovarian PDTO lines capture genomic and transcriptomic features of parental tumor. (**A**) Genome-wide heatmap of DNA copy number gains (blue) and losses (red) of tumor tissues and paired PDTO. A = Ascites-derived PDTO and T = Tumor–derived PDTO. (**B**) Distribution of tumor samples and paired PDTO on UMAP clusters based on gene expression profile following NMF (k = 12). * PDTO that do not have a matched tumor sample counterpart

For 15 PDTO and their matched tumor obtained between 2018 and 2021, low-pass WGS was performed in order to analyze copy number variation (CNV) across the genome. Overall, ovarian PDTO models displayed a good level of similarity with their original tumor (Fig. 2A). The similarity level of all paired samples varied between 0.4 and 1 with 60% of the pairs above 0.7 (Table S3).

Transcriptomic profiling was performed on the same paired samples (*n* = 15) and displayed a strong correlation between genes expressed by the PDTO and the tumor (R² ranging from 0.613 to 0.827 with a mean of 0.693) (Fig. S2A and B). However, Uniform Manifold Approximation and Projection (UMAP) visualization of all samples showed a clear split, the tumor samples clustering together away from PDTO samples (Fig. S2C). Analysis of differentially expressed gene enrichment showed a strong enrichment in immunology-related pathways, consistent with the fact that stromal cell component is lacking in PDTO models (Fig. S2D and E). In order to better compare paired samples, a non-negative matrix factorization was used. NMF decomposes gene expression profiles into combinations of latent components, referred to as transcriptional programs. Each sample is then represented by a weight vector indicating its contribution to each program. This dimensionality reduction allows for more robust comparisons across samples by focusing on dominant biological signals, while reducing the impact of noise. To assess the similarity between tumor samples and their matched PDTO, we performed both hierarchical clustering and unsupervised clustering analyses. The forced hierarchical clustering allowed us to highlight the overlap between tumor and PDTO samples, with most matched pairs having a close profile (Fig. S3A). In parallel, unsupervised clustering confirmed that the sample tend to cluster by pairs (Fig. S3B and C). Altogether, these analyses demonstrate that our heterogeneous panel of ovarian PDTO is broadly representative of their tumors of origin.

### PDTO drug response correlation to patient clinical outcome

**Fig. 3:**
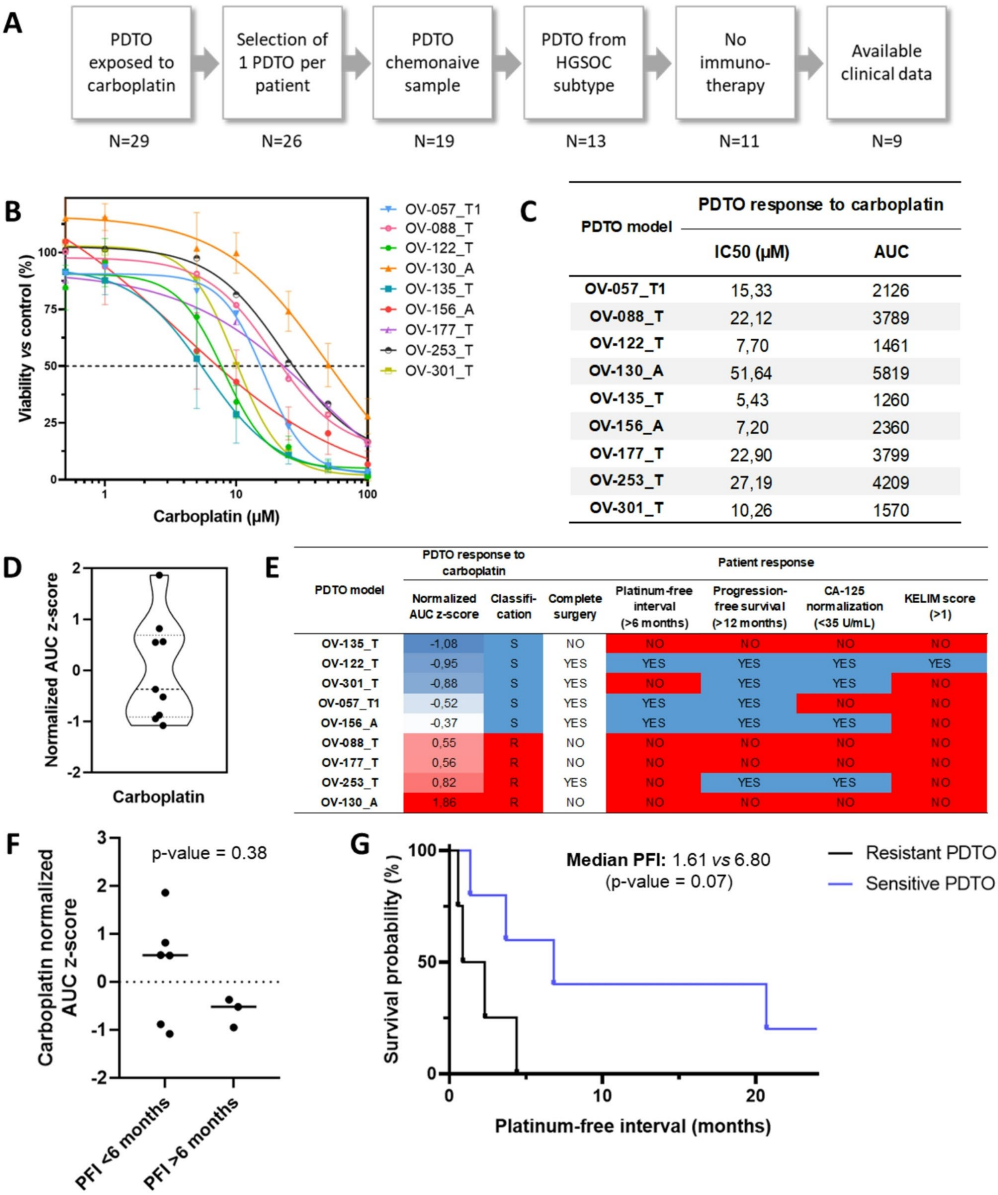
Ovarian PDTO lines recapitulate patient response to carboplatin. (**A**) Flow chart of the PDTO selection for the comparison with patient clinical outcome. (**B**) Dose-response curves of the 9 selected PDTO, each curve is representative of at least two independent experiments. A = Ascites-derived PDTO and T = Tumor–derived PDTO. (**C**) Summary of the IC50 (µM) and AUC obtained in B. for the 9 PDTO models. (**D**) Representation of the carboplatin normalized AUC z-score (*n* = 9). (**E**) Comparison of the PDTO response to carboplatin and clinical outcome, expressed as complete surgery, platinum-free interval (PFI), progression-free survival (PFS), CA-125 normalization and KELIM score. (**F**) Dot plots comparing PDTO response to carboplatin, expressed as normalized AUC z-score, and clinical outcome expressed as PFI. Data were analyzed using unpaired two-sided Mann–Whitney test. (**G**) Kaplan-Meier plot comparing PFI of the resistant PDTO group (*n* = 5) and the sensitive PDTO group (*n* = 4). Data were analyzed using Gehan-Breslow-Wilcoxon test

Next, we performed drug-response profiling on 29 ovarian PDTO for which a clinical follow-up of more than 12 months was available, representing 26 patients out of 31, in order to explore the potential of PDTO to recapitulate patient clinical response to chemotherapy. Since carboplatin is the standard-of-care for all ovarian cancers, all PDTO were exposed to increasing concentrations of this drug and response was assessed using viability assay. We selected a homogenous sample of subjects among our cohort of patients to provide a meaningful analyse. Therefore, only PDTO derived from chemonaive HGSOC patients were considered. Patients who received immunotherapy during the first line treatment were excluded, since they were part of clinical trials and no double-blind release was done (Fig. 3A). The selected PDTO (*n* = 9) displayed heterogenous response to carboplatin with IC50 (half inhibitory concentration) ranging from 5.43 to 51.64 µM and AUC (area under the curve) from 1260 to 5819 (Fig. 3B and C). Normalized AUC z-score of the PDTO response to carboplatin was then plotted as a violin diagram (Fig. 3D) showing a clear split between sensitive and resistant PDTO around the value 0. When compared to the patient clinical outcome, the PDTO response to carboplatin was associated to the patient platinum-free interval (PFI, time between the last carboplatin cycle and the relapse) (Fig. 3E). Some discrepancies were found when compared to the progression-free survival (PFS), to the overall survival (OS) and to the CA-125 normalization (nadir < 35U/mL) while no correlation at all were found using KELIM score (Fig. 3E). Interestingly the most resistant patient, OV-130_A with a PFI inferior of 1 month (therefore considered as platinum-refractory) was by far the most resistant PDTO to carboplatin (Fig. 3C, D and E). However, the difference of carboplatin normalized AUC between platinum-sensitive (PFI ≥ 6 months) and platinum resistant (PFI < 6 months) patients did not reach significance (*p*-value = 0.38) (Fig. 3F), notably due to low statistical power and the influence of the OV-135_T and the OV-301_T PDTO models. Both models displayed sensitive response to carboplatin while the patients were considered as platinum-resistant. Despite the initial good response of these patients during the first three cycles of chemotherapy (Fig. S4A and B), patients OV-135 and the OV-301 relapsed respectively from a distant mediastinal metastasis and a periaortic lymph node. For the patient OV-135, a PDTO model was also established from a chemonaive ascitic sample (OV-135_A), but it displayed the same sensitivity to carboplatin (Fig. S4C and D). Overall, the response of chemonaive HGSOC PDTO allowed to distinguishing the patients with resistant PDTO (*n* = 5) with a median PFI of 1.61 months from the patients with sensitive PDTO (*n* = 4) with a median PFI of 6.80 months (*p*-value = 0.06) (Fig. 3G).

Next, our PDTO panel was exposed to a larger number of drugs used in the clinical management of OC. To that end, we firstly adapted our protocols in order to work on a smaller number of PDTO per replicate, allowing an easier and faster screening. This optimization was performed from 96-well plate to 384-well plate, and required 4 times less PDTO (50 vs. 200) (Fig. S5A). This miniaturization approach was validated on 15 PDTO models and showed a strong correlation between both protocols (Spearman *r* = 0.83, *p* = 0.002) (Fig. S5B and C).

**Fig. 4:**
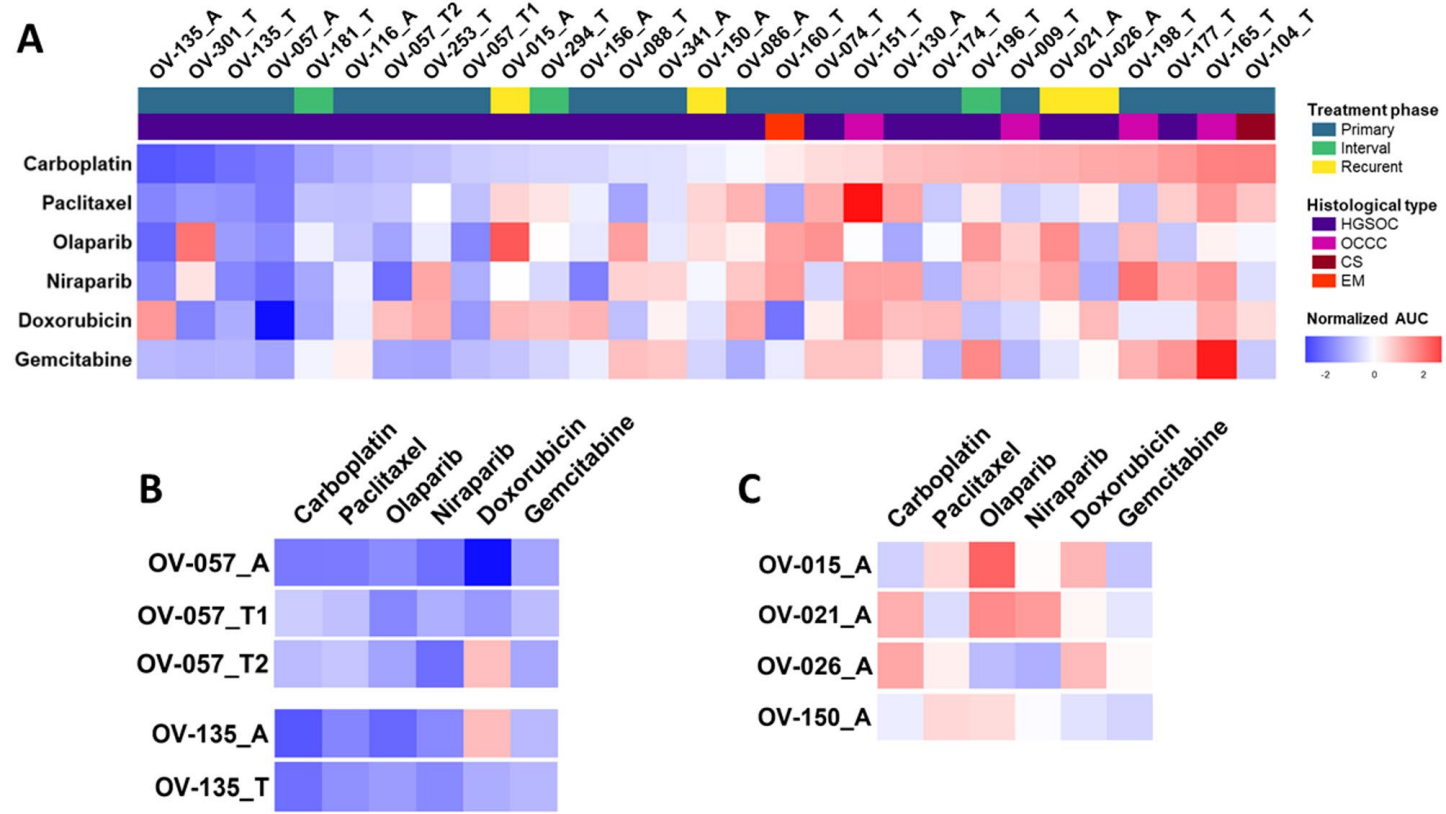
Drug screening of PDTO standard-of-care therapies in ovarian cancer. (**A**) Heatmap showing PDTO response to carboplatin, paclitaxel, olaparib, niraparib, doxorubicin and gemcitabine, expressed as normalized AUC z-score (*n* = 29 models). A = Ascites-derived PDTO and T = Tumor–derived PDTO. (**B**) Heatmap showing response of PDTO models derived from the same patient (*n* = 2 patients). (**C**) Heatmap showing response of PDTO models derived from recurrent ovarian cancer (*n* = 4 patients)

We then tested 6 different drugs, all routinely used in the clinical management of ovarian cancers (1st line, maintenance and following ones), namely, carboplatin, paclitaxel, doxorubicin, gemcitabine and 2 PARP inhibitors (olaparib and niraparib) on our panel of 29 ovarian PDTO (Fig. 4A). Globally, the sensitivity to carboplatin was associated to the sensitivity to other drugs, with some exceptions. For example, the PDTO model OV-301_T displayed a sensitivity to all drugs with the exception of the two PARPi. Conversely, the PDTO OV-130_A was resistant to all drugs except to olaparib (Fig. 4A).

As expected, all PDTO derived from OCCC and CS were in the platin-resistant side of the heatmap, consistent with the known platin-resistance of these subtypes (Fig. 4A).

We were able to derive multiple PDTO models from different tumor and ascitic samples of 2 patients. Regarding the patient OV-057, 3 chemonaives HGSOC PDTO models were established from an ascitic sample (OV-057_A), an epiploon node (OV-057_T1) and from a peritoneal node (OV-057_T2). 2 HGSOC PDTO models were also derived from an ascitic sample and from a peritoneal node of the patient OV-135 (OV-135_T and OV-135_A, respectively). Overall, PDTO models generated from the same patient responded similarly to all the drugs tested, whatever their origin (ascites or tumors) (Fig. 4B). However, it should be noticed that the response to doxorubicin present some discrepancies between models of the same patient, in both patients (Fig. 4B).

Interestingly, 4 PDTO models were established from 4 HGSOC patients with recurrence: patient OV-021 was sampled before the second line of treatment, patients OV-015 and OV-150 before the third line and patient OV-026 before the fourth line. All samples at recurrence were ascitic fluids (Table S2). The 4 recurrent PDTO displayed heterogenous response to the 6 drugs tested (Fig. 4C), with most of them on the spectrum of intermediate to resistant (Fig. 4A), probably due to the impact of the previous treatment lines.

### PDTO HR status determination

**Fig. 5:**
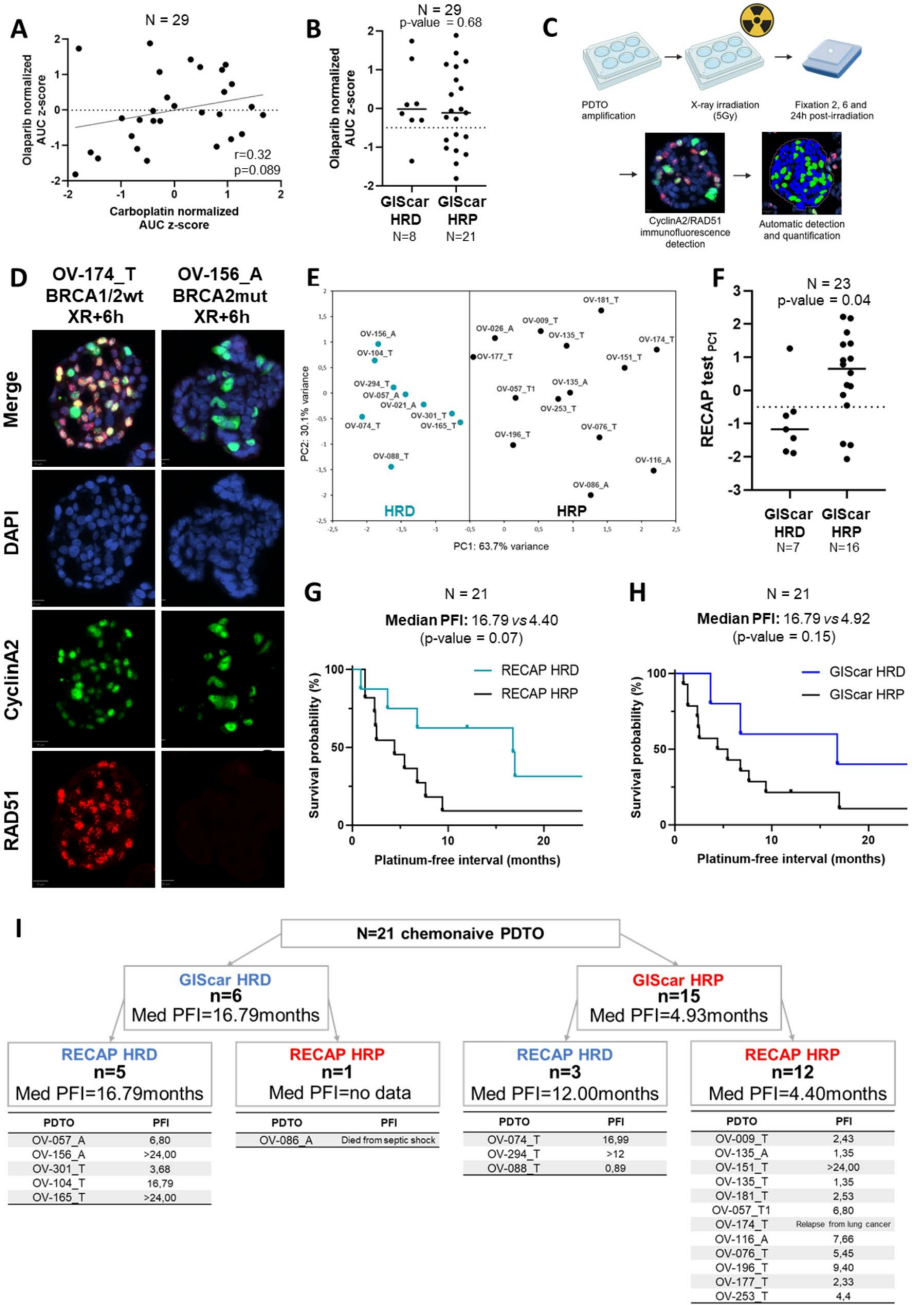
PDTO can be used to assess homologous recombination deficiency. (**A**) Correlation between the carboplatin and olaparib PDTO response, expressed as normalized AUC z-score (*n* = 29). (**B**) Dot plots comparing PDTO response to olaparib, expressed as normalized AUC z-score, and the genomic instability score HR status (*n* = 29). Data were analyzed using unpaired two-sided Mann–Whitney test. (**C**) Schematic representation of the RECAP test protocol. (**D**) DAPI staining to visualize nuclei (blue) and immunostaining of cyclinA2 (green) and RAD51 (red) in *BRCA1/2-*wt (OV-174_T) and *BRCA2-*mut (OV-156_A) PDTO models. (**E**) Principal Component Analysis (PCA) of RECAP test results (*n* = 23). A = Ascites-derived PDTO and T = Tumor–derived PDTO. (**F**) Dot plots comparing PDTO RECAP test PC1 and the genomic instability score HR status (*n* = 23). Data were analyzed using unpaired two-sided Mann–Whitney test. (**G**) Kaplan-Meier plot comparing PFI of the RECAP HRD group (*n* = 8) and the RECAP HRP group (*n* = 13). (**H**) Kaplan-Meier plot comparing PFI of the GIScar HRD group (*n* = 6) and the GIScar HRP group (*n* = 15). (**I**) Median PFI of OC patients from PDTO were derived depending on the HR status determined by GIScar analysis and RECAP test

Considering the importance of PARPi maintenance treatment for the therapeutic management of OC, we focused our attention on the evaluation of the potential use of PDTO for clinical decision making in this regard. It is commonly admitted that the platinum-sensitivity is one of the criteria of eligibility to PARPi. Therefore, the correlation between the PDTO response to carboplatin and to PARPi in our panel of 29 PDTO was evaluated. PDTO response to carboplatin and olaparib displayed a modest correlation (Spearman *r* = 0.32, *p* = 0.089) (Fig. 5A). Correlation between niraparib and carboplatin, olaparib and niraparib, were also tested and showed statistical significance (Spearman *r* = 0.57, *p* = 0.001 and Spearman *r* = 0.52, *p* = 0.004, respectively) (Fig. S6A and B). We then studied the correlation between the PDTO response to olaparib and HRD/HRP status of PDTO determined by the genomic instability score GIScar [8].

However, no correlation was observed between the GIScar analyses and the PDTO response to olaparib (Fig. 5B), neither to carboplatin (Fig. S6C) nor to niraparib (Fig. S6D). To complement this functional and genomic HRD assessment, we also summarized the main mutations detected in the PDTO cohort, including alterations in *TP53*, and homologous recombination repair–related genes (Fig. S7). Although the GIScar genomic instability score is derived from sequencing data of the same gene panel, no clear correlation was observed between specific punctual mutations in HR-related genes and HRD status as defined by GIScar or the RECAP test. A notable exception was the model OV-156_A harboring a *BRCA2* mutation, which was consistently classified as HRD by both approaches.

In an attempts to identify another functional assay, able to identify patients who could benefit from PARPi, we investigated the predictive potential of the RECAP test [27], and its automated analysis previously set up by our team [35]. Briefly, PDTO were exposed to X-ray and then grown for various times before being embedded in paraffin for immunofluorescence detection of RAD51 in CyclinA2 positive cells (Fig. 5C and D). Principal component analyses (PCA) distinguished two populations of PDTO models defined as HRD and HRP using a threshold of -0.5 on the PC1 axis (Fig. 5E). This analysis did not allow the establishment of a correlation between HR status and PDTO response to carboplatin, olaparib and niraparib (Fig. S6E, F and G). However, this RECAP-based HR status was correlated to the standard definition of HR status (genomic instability score, here defined by GISCar score) (*p*-value = 0.04) (Fig. 5F). However, the analysis of the PFI in this set of patients (after exclusion of 2 PDTO derived from samples obtained at recurrence) showed that RECAP HRD group (*n* = 8) had a median PFI of 16.79 months vs. 4.40 months in the RECAP HRP group (*n* = 13) (*p*-value = 0.07) (Fig. 5G). Similar values were obtained with GIScar score with a median PFI of 16.79 months vs. 4.92 months (*p*-value = 0.15) (Fig. 5H). These analyses have also been performed on HGSOC subgroup (*n* = 17) but are not conclusive (Fig. S6H and I). In addition, the RECAP test could bring supplementary information in the context in which genomic analyses lead to the conclusion of an HRP status (Fig. 5H). Indeed, in this arm (GIScar HRP, *n* = 15), our analysis of RECAP test reclassified 3 GIScar HRP as HRD. Interestingly, 2 of these 3 patients had a PFI superior to 12 months (median PFI = 12 months in GIScar HRP/RECAP HRD subgroup vs. 4.40 months in GIScar HRP/RECAP HRP subgroup).

## Discussion

We successfully established 37 long-term PDTO models from 31 patients with OC. The majority of the PDTO of the panel was derived from patient diagnosed with HGSOC, in accordance with the high frequency of this subtype observed among ovarian cancer patients (70% of all epithelial ovarian cancers) [2]. We were also able to produce PDTO models from rarer subtypes including OCCC, CS, MC and EM (for a total of 11 PDTO representing 8 patients). To our knowledge, this is the largest panel of PTDO from OCCC and CS although other teams had also been able to generate PDTO from such subtypes [19, 20, 36–38]. Knowing that most of these subtypes (especially OCCC and CS) are resistant to platinum-based chemotherapies, such preclinical models could be relevant to develop new therapeutic strategies for these tumors with few treatment options.

In this study, we achieved a long-term ovarian PDTO establishment rate (number of passages above 8) of 13.8%, showing that only a small proportion of OC patient could currently benefit from the PDTO-based precision medicine. However, this rate of establishment was difficult to compare with other studies since there is no consensus on the definition of a successful establishment of PDTO. In fact, many studies used different criteria of establishment and different definitions of long-term culture [23]. Overall, the success rate of long-term establishment (often defined as a number of passages above 4) varies from 18 to 53%, depending on the study considered [15, 22, 36, 37]. Interestingly, we observed notable differences in success rates between histological subtypes, with a particularly low efficiency in HGSOC (10%) compared to mucinous (100%), clear cell (25%), and carcinosarcoma (21.4%) subtypes. These differences may reflect distinct biological dependencies, including specific signaling pathways required for PDTO growth. Thus, it has been suggested that Wnt pathway activation may impair the growth of HGSOC-derived PDTO [15]. Although we tested a Wnt-depleted medium on a limited number of HGSOC samples (*n* = 3), we did not observe improved PDTO formation compared to our standard medium (data not shown). Nevertheless, the possibility remains that each histotype may require tailored culture conditions, highlighting the need to further investigate subtype-specific growth factor requirements to enhance PDTO line establishment efficiency. Senkowski et al. were able to improve their rate of establishment by systematically testing two different culture media to achieve long-term culture of PDTO from HGSOC in 53% of the cases, highlighting the importance of culture conditions [22]. In order to represent a larger proportion of OC patients, it would be necessary to work on earlier passages of PDTO. Indeed, as demonstrated by Bi et al., the use of PDTO during the first passages allowed to perform drug sensitivity assay for 83% of the tumor samples [38].

We characterized our PDTO panel with a set of tumor markers routinely used in OC diagnosis and PDTO showed a strong ability to reflect the features of their sample of origin in accordance with their histotypes. However, WT1 expression was reduced in some HGSOC-derived PDTOs compared to the corresponding tumors. Since WT1 expression is known to be regulated by TGF-β signalling [39, 40], this observation may be explained by the use of a TGF-β pathway inhibitor in our culture medium, as well as the absence of stromal cells in our model, which are partially responsible for TGF-β secretion. Genetic analyses also demonstrated a tight correlation between the CNV of the original tumor and that of paired PDTO. Thus, these analyses showed that PDTO not only recapitulate inter-patient heterogeneity, but also that our PDTO models are matched with their tumor of origin. Interestingly, intra-patient heterogeneity was retained as well, since PDTO from both tumor and ascitic samples of 2 patients (OV-057 and OV-135) maintained their original sample CNV profile. At the transcriptomics level, despite a strong correlation between the gene expression profile of the tumor sample and the paired PDTO, clustering showed that tumors were still closer to each other than to their PDTO mainly due to the lack of the stromal component. An alternative approach using non-negative matrix factorization allowed us to only consider the cancer component of paired samples and revealed that the PDTO recapitulate the transcriptomic profile of their tumor of origin in our relative long-term culture.

To assess whether ovarian PDTO could predict patient response to first-line chemotherapy, only chemonaive PDTO were selected, since PDTO from previously treated patients might have gained alterations that could introduce bias in response to treatments. We were able to match the PDTO response to carboplatin and the PFI of the patients following the first line treatment. Out of 9 tested patients, 2 displayed a difference between the clinical response and the paired PDTO sensitivity to carboplatin, whether PDTO were derived from carcinomatosis nodes or from ascitic samples. The medical history of these patients showed that, although they did not undergo complete surgical resection at the initial clinical management, both patients responded well to the first cycles of chemotherapy, as evidenced by the drastic decrease in CA-125 and the partial response assessed by mid-line RECIST. These observations suggested a response from the whole tumor mass, in accordance with the observations made on their PDTO. This shows that PDTO can predict tumor cell response to first-line carboplatin. However, in some cases of highly aggressive tumors or tumors showing a great plasticity, rapid recurrence may be observed despite a good initial clinical response, itself correlated with the PDTO response. For the concerned patients, the recurrence was detected in a mediastinal lesion (OV-135) or in a periaortic lymph node (OV-301), which may also suggest a sampling bias representing one of the limitations of these PDTO-based functional assays. Nevertheless, this limitation is also observed in all types of predictive assays (genomic, transcriptomic or functional assays), and the tumor heterogeneity in some patients may constitute an obstacle to the use of these functional assays for predictive purposes.

The main limitations of this study are the low establishment rate and the delay between the tumor/ascites sampling and the results of the predictive functional assay, making it currently not suitable in a context of clinical routine. In this study, the main objective was to assess the feasibility of establishing ovarian PDTO and to study their representativity of the patient at a genomic, transcriptomic and functional level in order to evaluate their predictive potential. We therefore established long-term PDTO, as they have the advantage of being stable and widely amplifiable for basic and translational applications. Regarding PDTO use for precision medicine, it becomes necessary to work on early passages despite small amount of PDTO, to be able to perform the functional assay rapidly and for most of the patients (around 90% according to Bi and al. [38]).

In this study, numerous PDTO models were established from ascitic samples (11/37) an integrative biological material of particular interest since ascites is present in 1/3 of ovarian cancer at the initial diagnosis and in a large majority of cases at the recurrence [41]. It would therefore enable a potential longitudinal assessment of patient response to treatment. Moreover, PDTO from ascites would also be usable for predicting response to treatment at recurrence, when surgery is rarely considered, thereby making difficult the obtention of tumor cells. Herein, we collected paired tumor/ascitic samples from two HGSOC patients. Interestingly, paired PDTO from these samples displayed a similar sensitivity to all the drugs tested with the exception of doxorubicin. More paired PDTO models derived at the same or different time of the clinical course are needed to conclude about the predictive potential of ascites-derived PDTO, as initiated by Arias-Diaz et al. [42]. Future studies will also need to investigate the ability of PDTO established from post-treatment samples (second and later lines of therapy) to provide effective guidance for therapeutic decisions in the management of patients in more complicated clinical situations, with fewer therapeutic options available, especially in the context of platinum-resistance recurrence.

While PDTO response to carboplatin showed a tight correlation with paclitaxel response, modest correlation were found between carboplatin and the two PARPi tested (olaparib and niraparib), in line with platinum sensitivity being suggested as a biomarker for predicting response to PARPi [43]. However, the PDTO response to PARPi or carboplatin was not correlated to HRD status determined either by GIScar or by our novel approach using the RECAP test. On the other hand, this RECAP test classification of HRP and HRD PDTO was closely correlated to the GIScar scoring as well as to the patient clinical response to first line chemotherapy defined by the PFI. Interestingly, despite this concordance between GIScar and RECAP, no clear association was found between GIScar status and the presence of mutations in HR-related genes. Given the established clinical relevance of GIScar, particularly its correlation with olaparib response in patients [8], this observation reinforces the idea that mutational profiling alone is not sufficient to define HRD status in a clinically actionable way. This is due not only to the potential absence of detectable mutations, but also to the complexity inherent to their interpretation, including the selection of relevant genes, the functional relationships between them, and the difficulty in translating such data into therapeutic decisions. These results also highlight the biological complexity of HRD, which may arise through multiple mechanisms beyond direct gene mutation (e.g., promoter methylation, gene silencing, protein mislocalization). By contrast, functional assays based on genomic scarring (such as GIScar) or dynamic evaluation of HR activity (such as the RECAP test) provide integrated information on the biological consequences of HRD, offering a more direct and clinically relevant basis for therapeutic guidance. Unfortunately, only a very small number of patients enrolled in this study were prescribed PARPi during their clinical management, making it impossible to establish a correlation between functional assays and the patient clinical response to PARPi. Both GIScar scores and functional PDTO responses appear to have some predictive value when considered separately: GIScar scoring is closely correlated with the patient response to olaparib [8], and PDTO response to carboplatin in our cohort was generally consistent with clinical outcomes. However, the absence of correlation between these two approaches in our dataset raises important biological and methodological questions. Several non-exclusive explanations may underlie this observation. First, the limited number of models available reduces the statistical power and potentially impairs our ability to detect a meaningful association. Second, GIScar and functional PDTO assays likely capture distinct and complementary biological dimensions: GIScar reflects a static, cumulative signature of HRD, while PDTO response provides a dynamic measure of cellular sensitivity to drug-induced damage, potentially influenced by additional mechanisms such as drug detoxification, efflux, or cell cycle regulation. Notably, two PDTO OV-021_A and OV-301_T showed marked resistance to olaparib while they were classified as HRD by GIScar. Third, although GIScar was computed directly on DNA extracted from PDTO, the threshold used to define HRD versus HRP status was derived from patient tumor cohorts, in the context of olaparib treatment in OC. In the absence of sufficient numbers to recalibrate a PDTO-specific cut-off, applying this threshold to PDTO may have introduced classification bias and weakened concordance with functional outcomes. Fourth, the nature of the DNA damage induced by different agents must be considered: carboplatin generates DNA adducts and crosslinks, while RECAP test and GIScar scoring are based on sensitivity to ionizing radiation and the genomic scars it leaves, respectively, potentially reflecting different repair mechanisms. Finally, the readout used in our PDTO assays — based on short-term viability — may lack sensitivity to detect long-term survival or self-renewal potential after treatment. Alternative assays, such as organoid-forming efficiency post-treatment [44], could provide a more clinically relevant measure of therapeutic response. Together, these elements highlight the complexity of modeling treatment sensitivity in vitro and suggest that integrating multiple parameters (genomic, functional, etc…) may be required to improve predictive accuracy in future studies.

Furthermore, our study suggests that testing the functionality of the HR pathway based on the RECAP test could offer superior performance to the genomic test. Indeed, within the group of patients identified as HRP by GIScar, the patients confirmed HRP by the RECAP test showed a median PFI of 4.4 months while the patients identified as HRD by the RECAP test had a median PFI of 12 months (Fig. 5I). In this this latter group, 1 of the 3 patients concerned appears to be resistant to treatment (PFI = 0.89). However, the other 2 patients have PFIs of 16.98 and > 12 months, strongly suggesting that the RECAP test has classified them correctly. Thus, RECAP test could identify HRD patients who had not been previously identified using genomics and enabling them to benefit from PARPi.

## Conclusion

In conclusion, our study shows the feasibility and the interest of generating ovarian PDTO of different histotypes to predict response of first line treatment, and highlights the potential of functional assays to complement the genomic tests currently used for PARPi prescription. Although our cohort has its limitations (small size and heterogeneity of clinical parameters), it is one of the largest collections described to date. This study also highlights the many challenges that still need to be met before the routine use of these assays in clinical oncology. This includes the necessity of shortening the time needed to assess response to treatment, reducing the quantity of material required to perform these functional assays, and increasing the success rate of PDTO culture. It is thus essential that future investigations focus on the application of functional assays as soon as possible, ideally just after the formation of the first PDTO (early passages). This will involve to determine their ability to summarize the characteristics and heterogeneity of the tumors from which they are derived, and will require the development of miniaturization and standardization of functional assays. It will also be necessary to compare PDTO results to functional assays whether they are used in early passage or after long-term establishment. The presence of stromal cells in the first passages is also likely to modify response to treatment. This may be an advantage, but must be monitored to ensure sufficient robustness to enable these tests to be applied to a precision medicine approach in ovarian cancers. All the work being carried out by the community on these burning issues is opening up both interesting prospects and new questions, but it is only if they are resolved that PDTO will find their full potential in the management of these cancers.

## Supplementary Information

Below is the link to the electronic supplementary material.

Supplementary Material 1

Supplementary Material 2

## Data Availability

CNA and RNA-seq data of the current study are available in the Sequence Read Archive repository (accession no. PRJNA1222342). All other data supporting the findings of this study are available from the corresponding authors on reasonable request. All materials will be available upon request through a material transfer agreement.

## Abbreviations

AUC: Area Under the Curve
BRCA1/2: Breast Cancer genes 1 and 2
BSA: Bovine Serum Albumin
CA-125: Cancer Antigen 125
CNV: Copy Number Variation
CS: Carcinosarcoma
DAB: 3,3′-Diaminobenzidine
DAPI: 4′,6-Diamidino-2-Phenylindole
DI: Damage Index
EM: Endometrioid
FFPE: Formalin-Fixed Paraffin-Embedded
FIGO: International Federation of Obstetrics and Gynecology
FITC: Fluorescein Isothiocyanate
GIS: Genomic Instability Score
GIScar: Genomic Instability Scar (academic HRD test)
GMM: Gaussian Mixture Model
HE: Hematoxylin and Eosin
HGSOC: High-Grade Serous Ovarian Cancer
HR: Homologous Recombination
HRD: Homologous Recombination Deficient
HRP: Homologous Recombination Proficient
IC50: Half Maximal Inhibitory Concentration
MC: Mucinous Carcinoma
NMF: Non-Negative Matrix Factorization
OBM: Organoid Basal Medium
OC: Ovarian Cancer
OCCC: Ovarian Clear Cell Carcinoma
OS: Overall Survival
PARPi: Poly(ADP-ribose) Polymerase Inhibitor
PCA: Principal Component Analysis
PDTO: Patient-Derived Tumor Organoid
PFI: Platinum-Free Interval
PFS: Progression-Free Survival
RDI: Radio-induced Damage Index
RECAP: REpair CAPacity
RECIST: Response Evaluation Criteria In Solid Tumors
RI: Reparation Index
STR: Short Tandem Repeat
UMAP: Uniform Manifold Approximation and Projection
VAF: Variant Allele Frequency
