# Supplementary material for: Long-term patient-derived ovarian cancer organoids closely recapitulate tumor of origin and clinical response
Source: J Exp Clin Cancer Res. 2025 Oct 7;44:282. doi: 10.1186/s13046-025-03537-x (PMC12502492; doi:10.1186/s13046-025-03537-x)
Supplement: Supplementary file 2 — Supplementary Material 2 [file 13046_2025_3537_MOESM2_ESM.pdf]

Supplemental data

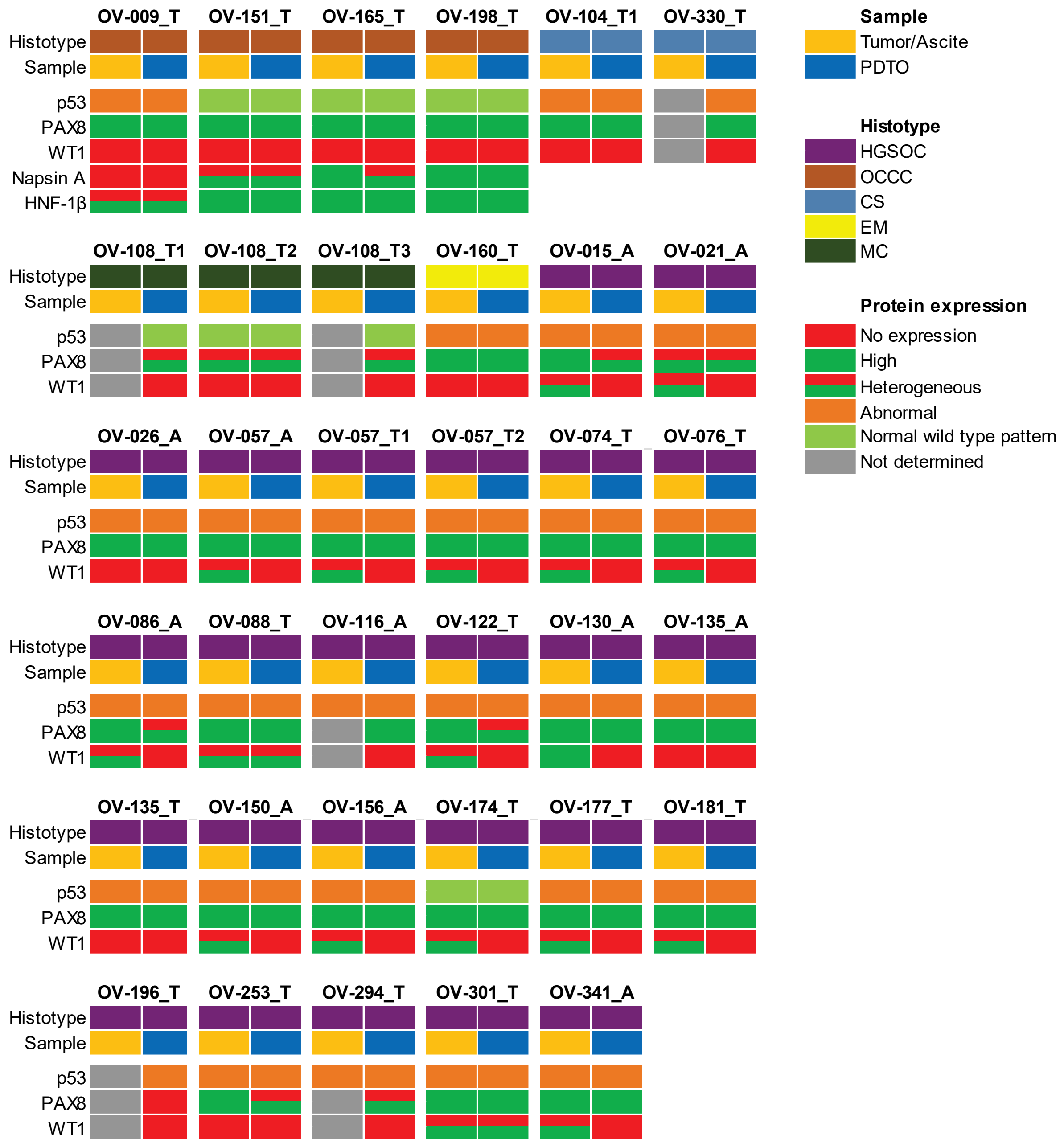

Fig. S1. Immunohistochemical profiles of key diagnostic markers in both tumor samples and their matched PDTO. Each rectangle represents the expression level of a given marker (PAX8, WT1, P53, Napsin A, HNF1β) in either the PDTO or the paired tumor sample. Expression levels are color-coded as follows: green = strong expression, red = no expression, red and green = heterogeneous expression within the sample. For p53 staining, expression was dichotomized into "abnormal" (overexpression, complete absence, or cytoplasmic pattern) (orange) and “normal wild type pattern” (light green). Grey boxes indicate that IHC could not be performed due to the absence of tumor material or cancer cells in tumor sample.

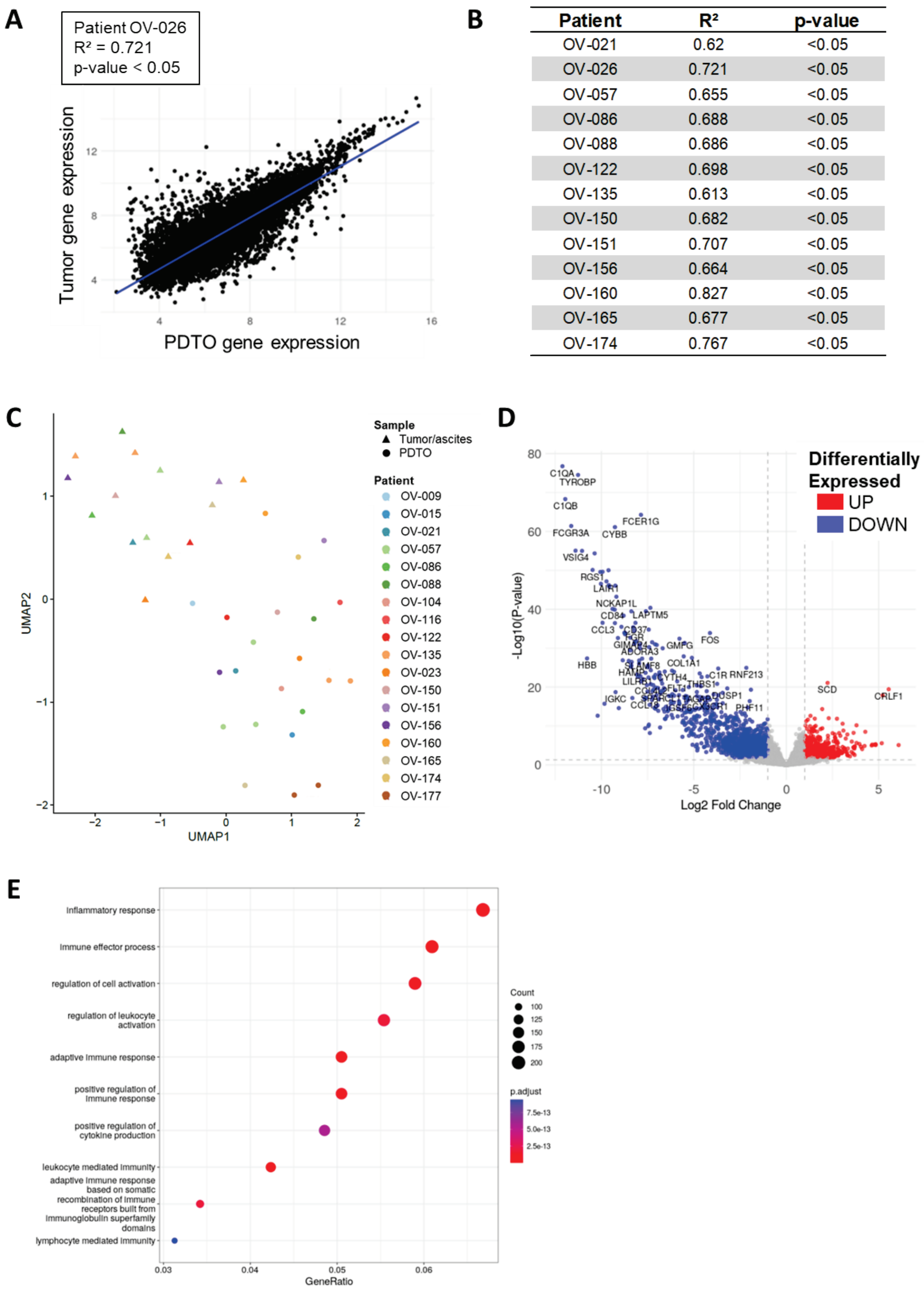

Patient	R^2	p-value
OV-021	0.62	<0.05
OV-026	0.721	<0.05
OV-057	0.655	<0.05
OV-086	0.688	<0.05
OV-088	0.686	<0.05
OV-122	0.698	<0.05
OV-135	0.613	<0.05
OV-150	0.682	<0.05
OV-151	0.707	<0.05
OV-156	0.664	<0.05
OV-160	0.827	<0.05
OV-165	0.677	<0.05
OV-174	0.767	<0.05

Fig. S2. Transcriptomics analysis distinguishes tumor from PDTO samples. (**A**) Scatter plot of the tumor and paired PDTO gene expression for the patient OV-023 with the Spearman's correlation. (**B**) Spearman's correlation coefficients of the tumor and paired PDTO gene expression for each patient. (**C**) Distribution of tumor samples and paired PDTO on Uniform Manifold Approximation and Projection (UMAP) clusters based on gene expression profile. (**D**) Volcano plot of differentially expressed genes between tumor samples and paired PDTO. Up- and down-regulated genes are represented in red and blue, respectively. (**E**)

Functional enrichment analysis of biological processes (BP) by Over-Representation Analysis (ORA) of differentially expressed genes between tumor samples and paired PDTO.

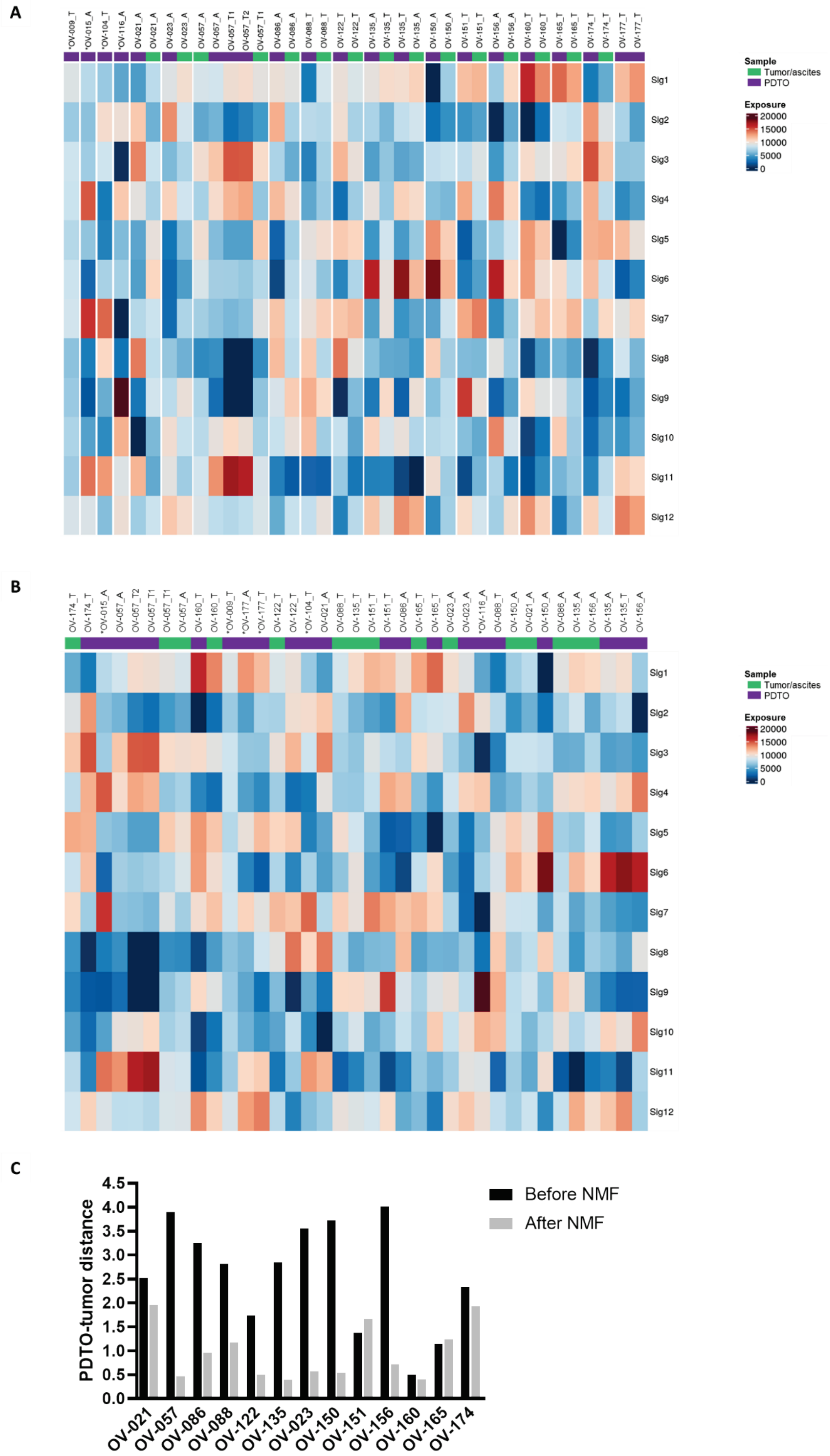
A
*OV-009_T
*OV-015_A
*OV-104_T
*OV-116_A
OV-021_A
OV-021_A
OV-023_A
OV-023_A
OV-057_A
OV-057_A
OV-057_T1
OV-057_T2
OV-057_T1
OV-086_A
OV-086_A
OV-088_T
OV-088_T
OV-122_T
OV-122_T
OV-135_A
OV-135_T
OV-135_T
OV-135_A
OV-150_A
OV-150_A
OV-151_T
OV-151_T
OV-156_A
OV-156_A
OV-160_T
OV-160_T
OV-165_T
OV-165_T
OV-174_T
OV-174_T
OV-177_T
OV-177_T
Sig1
Sig2
Sig3
Sig4
Sig5
Sig6
Sig7
Sig8
Sig9
Sig10
Sig11
Sig12
Sample
Tumor/ascites
PDTO
Exposure
20000
15000
10000
5000
0
B
OV-174_T
OV-174_T
*OV-015_A
OV-057_A
OV-057_T2
OV-057_T1
OV-057_T1
OV-057_A
OV-160_T
OV-160_T
*OV-009_T
*OV-177_A
*OV-177_T
OV-122_T
OV-122_T
*OV-104_T
OV-021_A
OV-088_T
OV-135_T
OV-151_T
OV-151_T
OV-086_A
OV-165_T
OV-165_T
OV-023_A
OV-023_A
*OV-116_A
OV-088_T
OV-150_A
OV-021_A
OV-150_A
OV-086_A
OV-135_A
OV-156_A
OV-135_A
OV-135_T
OV-156_A
Sig1
Sig2
Sig3
Sig4
Sig5
Sig6
Sig7
Sig8
Sig9
Sig10
Sig11
Sig12
Sample
Tumor/ascites
PDTO
Exposure
20000
15000
10000
5000
0
C
PDTO-tumor distance
4.5
4.0
3.5
3.0
2.5
2.0
1.5
1.0
0.5
0.0
OV-021
OV-057
OV-086
OV-088
OV-122
OV-135
OV-023
OV-150
OV-151
OV-156
OV-160
OV-165
OV-174
Before NMF
After NMF

Fig. S3. Data dimension reduction brings gene expression profiles of tumors closer to those of their paired PDTO. (**A**) Forced and (**B**) unsupervised hierarchical clustering and heatmaps of exposure values of PDTO and tumor samples, based on non-negative matrix factorization (k=12) of RNA-seq expression data. (**C**) Histogram showing distribution of Euclidean distances between tumor samples and their paired PDTO using their UMAP coordinates, before and after performing NMF(k=12).

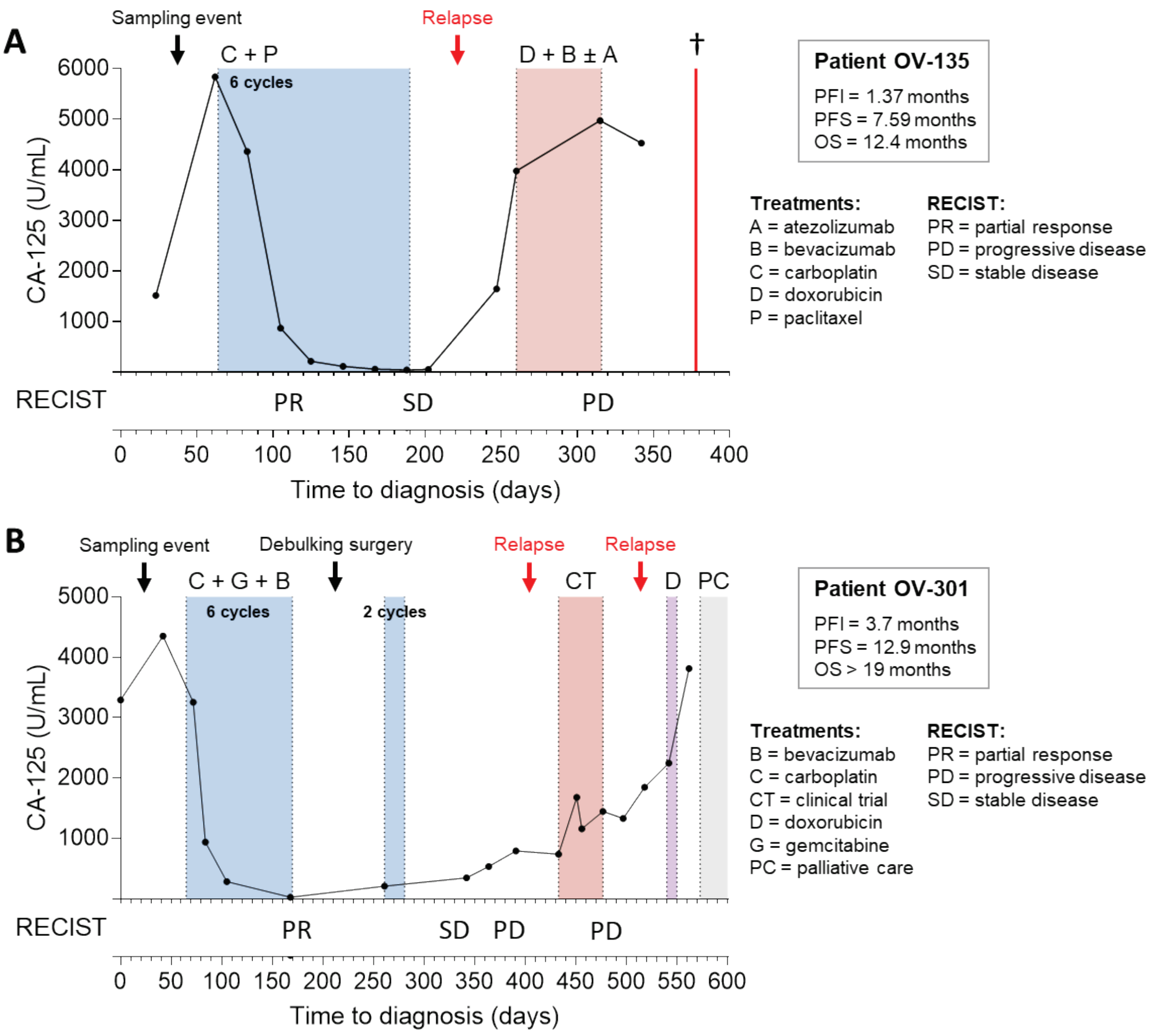

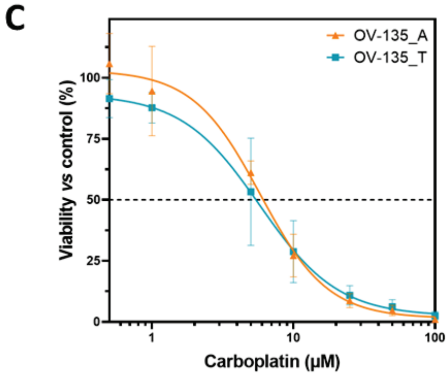

D

PDTO model	PDTO response to carboplatin	
	IC50 (µM)	AUC
OV-135_A	6,12	1162
OV-135_T	5,43	1260

Fig. S4. Detailed clinical response of patient OV-135 and OV-301. (**A**) Patient OV-135 response to 1st and 2nd line treatment over time, expressed as serum level of Cancer Antigen 125 (CA-125), RECIST evaluation as well as PFI, PFS and OS. (**B**) Patient OV-301 response to 1st, 2nd line and 3rd line treatment over time, expressed as serum level of Cancer Antigen 125 (CA-125), RECIST evaluation as well as PFI, PFS and OS. (**C**) Dose-response curves of the 2 PDTO models OV-135_A and OV-135_T to carboplatin. Each curve is the representative of two independent experiments. (**D**) Summary of the IC50 (µM) and AUC obtained in (**B**).

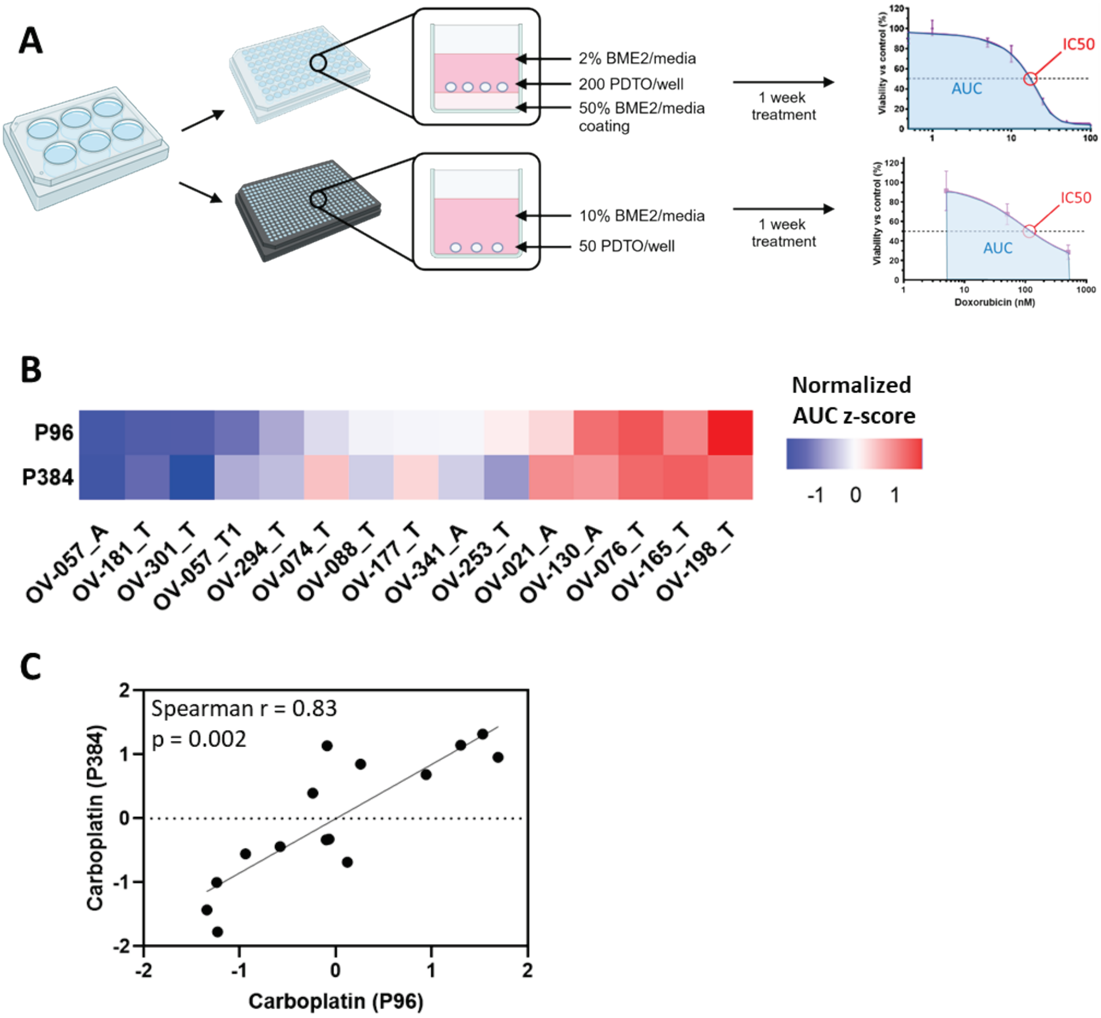

Fig. S5. PDTO treatment protocol adaptation from 96-well plate to 384-well plate. (**A**) Schematic representation of PDTO treatment protocols in 96- or 384-well plate. (**B**) Heatmap comparing the response of PDTO to carboplatin, expressed as normalized AUC z-score, in 96- and in 384-well plates (n=15). A = Ascites-derived PDTO and T = Tumor–derived PDTO. (**C**) Scatter plot of the response of PDTO to carboplatin, expressed as normalized AUC z-score, in 96- and in 384-well plate (n=15) with the Spearman's correlation.

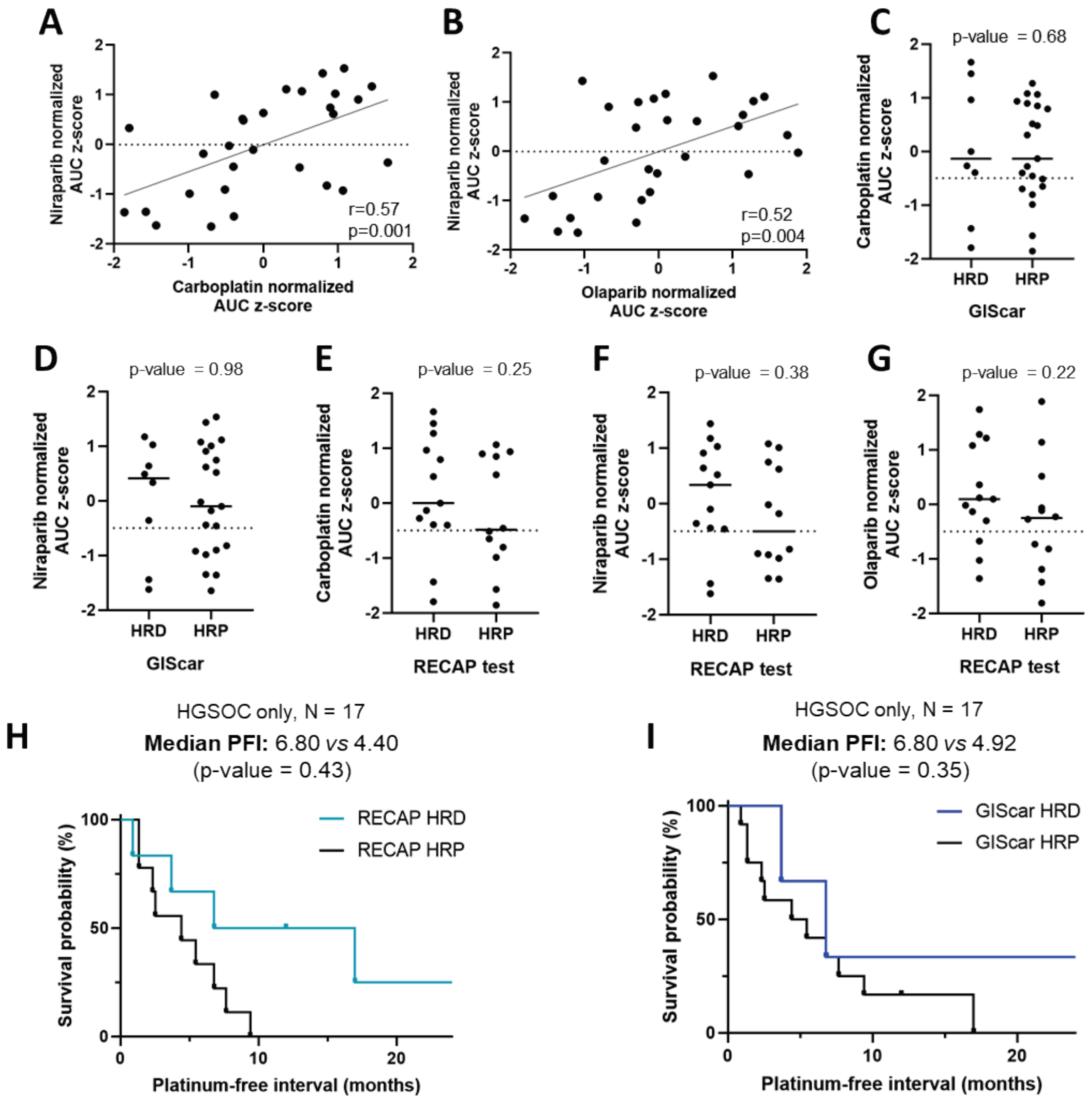

Fig. S6. PDTO can be used to assess homologous recombination deficiency. (**A**) Scatter plot of carboplatin and niraparib normalized AUC z-scores with the Spearman's correlation (n=29). (**B**) Scatter plot of olaparib and niraparib normalized AUC z-scores with the Spearman's correlation (n=29). (**C, D**) Dot plots comparing PDTO response to (**C**) carboplatin or (**D**) niraparib, expressed as normalized AUC z-score, and the genomic instability score HR status (n=29). Data were analyzed using unpaired two-sided Mann–Whitney test. (**E, F, G**) Dot plots comparing PDTO response to (**E**) carboplatin, (**F**) niraparib, or (**G**) olaparib, expressed as normalized AUC z-score, and the RECAP test HR status (n=23). Data were analyzed using unpaired two-sided Mann–Whitney test. (**H**) Kaplan-Meier plot comparing PFI of the RECAP HRD group (n=6) and the RECAP HRP group (n=11). (**I**) Kaplan-Meier plot comparing the PFI of the GIScar HRD group (n=4) and the GIScar HRP group (n=13).

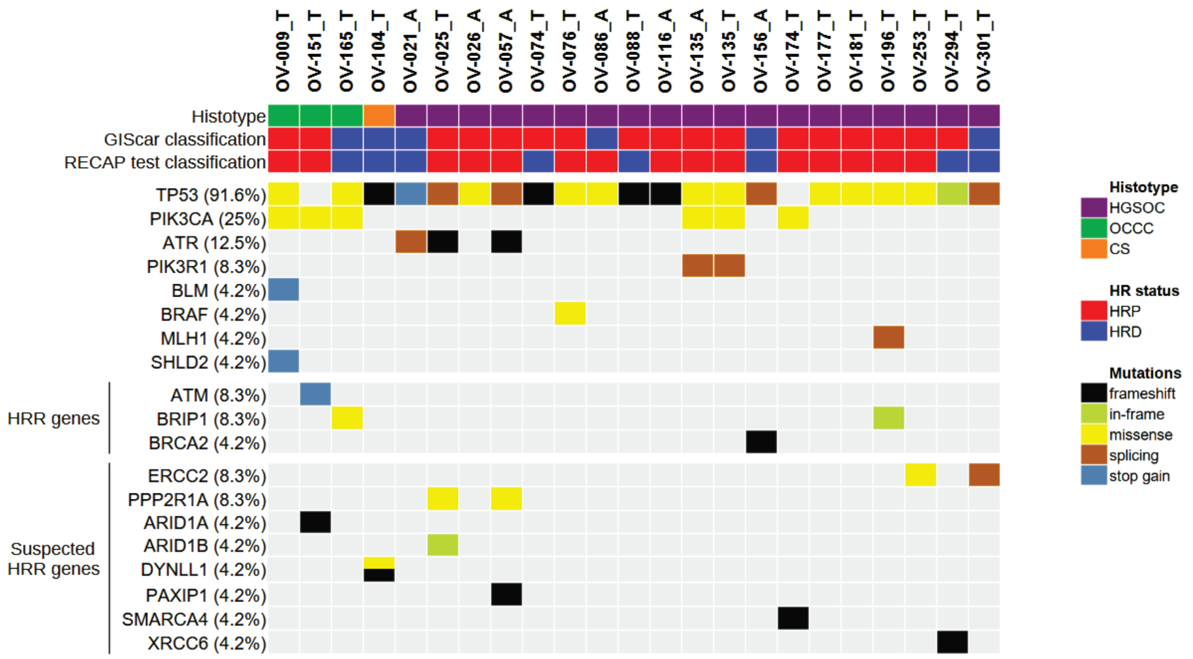

Fig. S7. Mutations in long-term patient-derived ovarian cancer organoids. Summary of mutations identified in PDTO by deep targeted sequencing, together with their percentage representation in this dataset. HRR: homologous recombination repair.

Histotypes	RECEIVED AT THE LABORATORY		SHORT TERM ESTABLISHMENT (>p3) ESTABLISHED		SHORT TERM ESTABLISHMENT (>p3) % OF ESTABLISHMENT		LONG TERM ESTABLISHMENT (>p8) ESTABLISHED		LONG TERM ESTABLISHMENT (>p8) % OF ESTABLISHMENT	
	Number of samples	Number of patients represented	Number of samples	Number of patients represented	% of samples	% of patients represented	Number of samples	Number of patients represented	% of samples	% of patients represented
**AGC**	15	9	1	1	6,7%	11,1%	0	0	0,0%	0,0%
**CCC**	16	13	5	5	31,3%	38,5%	4	4	25,0%	30,8%
**CS**	14	10	4	5	28,6%	50,0%	3	2	21,4%	20,0%
**EM**	18	10	5	4	27,8%	40,0%	1	1	5,6%	10,0%
**HGSOC**	261	170	50	40	19,2%	23,5%	26	23	10,0%	13,5%
**HSCC**	1	1	0	0	0,0%	0,0%	0	0	0,0%	0,0%
**LGSOC**	20	9	7	5	35,0%	55,6%	0	0	0,0%	0,0%
**LSCC**	1	1	0	0	0,0%	0,0%	0	0	0,0%	0,0%
**MC**	3	1	3	1	100,0%	100,0%	3	1	100,0%	100,0%
**TOTAL**	**349**	**224**	**75**	**61**	**21,5%**	**27,2%**	**37**	**31**	**10,6%**	**13,8%**

AGC: adult granulosa cell tumor
CCC: clear cell carcinoma
CS: carcinosarcoma
EM: endometroid carcinoma
HGSOC: High-grade serous ovarian carcinoma
HSCC: hypercalcemic small cell carcinoma
LGSOC: low-grade serous ovarian carcinoma
LSCC: lung-type small cell carcinoma
MC: mucinous carcinoma

Table S1. PDTO histotypes diversity and short- and long-term establishment rates.

PDTO information		Clinical information			Clinical outcome			Treatment lines						
PDTO model	Sample timing	Histotype	FIGO stage	Complete surgery	PFI (months)	PFS (months)	OS (months)	1st line treatment	1st line maintenance	2nde line treatment	2nde line maintenance	3rd line treatment	4th line treatment	5th line treatment
OV-009_T	Initial	CCC	4	Yes - Initial	2,43	8,08	15,31	Carboplatin + paclitaxel		Doxorubicin		Gemcitabine	Radiotherapy	
OV-015_A	Recurrent 3rd line	HGSOC	4	No	2,99	10,25	17,41	Carboplatin + paclitaxel	Bevacizumab	Paclitaxel + pazopanib		Gemcitabine	Doxorubicin	
OV-021_A	Recurrent 2nd line	HGSOC	3	No	6,41	10,64	22,11	Carboplatin + paclitaxel	Bevacizumab	Carboplatin + doxorubicin + bevacizumab ± atezolizumab	Bevacizumab ± atezolizumab	Gemcitabine		
OV-026_A	Recurrent 4th line	HGSOC	4	No	15,93	21,42	39,49	Carboplatin + paclitaxel + bevacizumab	Bevacizumab	Carboplatin + doxorubicin		Paclitaxel	Gemcitabine	
OV-057_T2	Initial	HGSOC	3	Yes - Interval	6,80	14,23	21,49	Carboplatin + paclitaxel	Bevacizumab	Carboplatin + gemcitabine		Paclitaxel		
OV-057_T1	Initial													
OV-057_A	Initial													
OV-074_T	Initial	HGSOC	3	Yes - Initial	16,99	23,10	32,89	Carboplatin + paclitaxel + bevacizumab ± TSR042	Bevacizumab + niraparib	Carboplatin + doxorubicin		Gemcitabine		
OV-076_T	Initial	HGSOC	4	No	5,45	10,55	18,79	Carboplatin + paclitaxel + bevacizumab ± TSR042	Bevacizumab + niraparib	Paclitaxel				
OV-086_A	Initial	HGSOC	3	No	Died from septic shock		2,83	Carboplatin + paclitaxel						
OV-088_T	Initial	HGSOC	4	No	0,89	6,37	8,28	Carboplatin + paclitaxel + bevacizumab	Bevacizumab	Doxorubicin				
OV-104_T1	Initial	CS	3	Yes - Initial	16,79	22,11	22,70	Carboplatin + paclitaxel + bevacizumab ± TSR042	Bevacizumab + niraparib	Carboplatin + doxorubicin				
OV-104_T2	Initial													
OV-108_T1	Initial	MC	1	Yes - Initial	>43	>50	>50	Carboplatin + paclitaxel						
OV-108_T2	Initial													
OV-108_T3	Initial													
OV-116_A	Initial	HGSOC	4	No	7,66	13,11	19,55	Carboplatin + paclitaxel + bevacizumab ± TSR042	Bevacizumab + niraparib	Carboplatin				
OV-122_T	Initial	HGSOC	4	Yes - Initial	20,04	27,17	NC	Carboplatin + paclitaxel	Bevacizumab + olaparib	Carboplatin + doxorubicin		Paclitaxel		
OV-130_A	Initial	HGSOC	3	No	0,56	8,31	36,80	Carboplatin + paclitaxel	Bevacizumab	Carboplatin + gemcitabine	Niraparib	Paclitaxel		
OV-135_A	Initial	HGSOC	3	No	1,35	7,59	12,42	Carboplatin + paclitaxel		Doxorubicin + bevacizumab ± atezolizumab				
OV-135_T	Initial													
OV-150_A	Recurrent 3rd line	HGSOC	4	Yes - Interval	22,05	28,16	51,00	Carboplatin + paclitaxel	Bevacizumab	Carboplatin + radiotherapy	Niraparib	Paclitaxel		
OV-151_T	Initial	CCC	1	Yes - Initial	>36	>42	>42	Carboplatin						
OV-156_A	Initial	HGSOC	3	Yes - Closing	>32	>40	>40	Carboplatin + paclitaxel	Bevacizumab + olaparib					
OV-160_T	Initial	EM	3	Yes - Initial	>34	>40	>40	Carboplatin + paclitaxel ± pembrolizumab						
OV-165_T	Initial	CCC	1	Yes - Initial	>31	>37	>37	Carboplatin + paclitaxel						
OV-174_T	Initial	HGSOC	4	No	Relapsed from lung cancer		18,89	Carboplatin + paclitaxel + bevacizumab	Bevacizumab + erlotinib					
OV-177_T	Initial	HGSOC	4	No	2,33	6,97	29,57	Carboplatin + paclitaxel	Bevacizumab	Doxorubicin + bevacizumab		Paclitaxel	Carboplatin + paclitaxel	Letrozole
OV-181_T	Interval	HGSOC	4	No	2,53	8,90	11,89	Carboplatin + paclitaxel		Doxorubicin				
OV-196_T	Interval	HGSOC	3	Yes - Interval	9,40	16,76	17,61	Carboplatin + paclitaxel	Bevacizumab					
OV-198_T	Initial	CCC	3	Yes - Initial	1,61	8,48	>35	Carboplatin + paclitaxel		Doxorubicin + bevacizumab	Bevacizumab			
OV-253_T	Initial	HGSOC	4	Yes - Closing	4,40	13,57	>23	Carboplatin + paclitaxel + bevacizumab	Bevacizumab	Doxorubicin		Paclitaxel		
OV-294_T	Interval	HGSOC	3	Yes - Interval	>12	>18	>18	Carboplatin + paclitaxel	Bevacizumab					
OV-301_T	Initial	HGSOC	4	Yes - Closing	3,68	12,91	>17	Carboplatin + gemcitabine + bevacizumab	Bevacizumab	RGN4018 + cemiplimab		Doxorubicin		
OV-341_A	Initial	HGSOC	3	No	>7	>12	>12	Carboplatin + paclitaxel	Bevacizumab					
OV-330_T	Interval	CS	3	Yes - Interval	0,56	4,79	>14	Carboplatin + paclitaxel		Doxorubicin + bevacizumab				
OV-342_T	Initial	HGSOC	3	No	Died during the first carboplatin cycle		2,00	Carboplatin						

Table S2. Patient clinical information. CCC: clear cell carcinoma, CS: carcinosarcoma, EM: endometroid carcinoma, HGSOC: high-grade serous ovarian carcinoma, MC: mucinous carcinoma.

Patient and paired PDTO sample name	Histotype	Dice-Sørensen coefficient
**OV-021_A**	HGSOC	0,4
**OV-026_A**	HGSOC	0,9
**OV-057_T1**	HGSOC	0,5
**OV-057_A**	HGSOC	0,9
**OV-086_A**	HGSOC	0,7
**OV-088_T**	HGSOC	0,5
**OV-122_T**	HGSOC	0,5
**OV-135_T**	HGSOC	0,5
**OV-135_A**	HGSOC	0,9
**OV-150_A**	HGSOC	0,9
**OV-151_T**	CCC	1
**OV-156_A**	HGSOC	0,8
**OV-160_T**	EM	1
**OV-165_T**	CCC	1
**OV-174_T**	HGSOC	0,4

CCC: clear cell carcinoma

CS: carcinosarcoma

EM: endometroid carcinoma

HGSOC: High-grade serous ovarian carcinoma

Table S3. Copy-number variation (CNV) homology between paired samples.
